# *In vitro* reconstitution implicates multiple OMP assembly factors in CdiA^EC93^ toxin delivery

**DOI:** 10.1016/j.jbc.2026.113512

**Published:** 2026-09-22

**Authors:** Ana Katrina Y. Tiu, Haley E. Murphy, Gabrielle M. Beal, Christine L. Hagan

**Affiliations:** Department of Chemistry, The College of the Holy Cross, Worcester, Massachusetts, USA

**Keywords:** contact-dependent inhibition, gram-negative bacteria, bacterial toxin, outer membrane, protein translocation, Bam complex, chaperone, membrane reconstitution

## Abstract

Contact-dependent inhibition is a mechanism of interbacterial competition in gram-negative bacteria. A large cell surface protein called CdiA is the key component of CDI systems; it inhibits the growth of neighboring, nonsibling bacteria by delivering a protein toxin through their cell envelopes. Here, we describe an *in vitro* reconstitution of the initial steps of the toxin delivery process, which normally occur in the outer membrane of a target cell. We show that a large, purified fragment of a CdiA protein from *Escherichia coli* EC93 spontaneously assembles into proteoliposomes containing the target cell receptor, BamA. BamA normally performs the essential function of assembling β-barrel proteins in the outer membrane using a lateral gate in its own β-barrel, but our data indicate that the CdiA assembly process does not depend on BamA’s gating action. Rather, CdiA assembles into the lipid bilayer once it has formed a stable association with the receptor. Our data further indicate that CdiA’s relatively rapid insertion into the membrane is followed by a slower autoproteolytic cleavage of the toxin domain at its C-terminal end. The periplasmic chaperones SurA and Skp increase the rate of that toxin cleavage process *in vitro*, and SurA overexpression *in vivo* sensitizes cells to CdiA intoxication. Collectively, our results suggest that CdiA may co-opt multiple factors in the β-barrel assembly pathway to facilitate efficient delivery of its toxin into target cells.

Gram-negative bacteria deploy a variety of protein toxins to kill competing bacteria in their environments (1, 2, 3). Many of these toxins have enzymatic or pore-forming activities that disrupt intracellular pathways; accordingly, the toxins must be transported through the outer membranes (OMs) of their targets—and often through their inner membranes (IMs) as well. Different toxin delivery systems provide the driving force those membrane translocation steps in different ways. For example, colicins are secreted proteins that are released by their producers into the surrounding environment (4). They then bind to receptors in the OMs of their targets and co-opt endogenous systems within those cells, which transduce energy from the IM’s electrochemical potential to the OM to import the toxin (5, 6, 7). Type VI secretion systems generate the mechanical force necessary to drive their toxins through the membranes of their targets by assembling massive needle-like structures within the producer bacteria (8, 9). A rapid contraction of a protein sheath that surrounds the needle then propels it through the producer’s cell envelope and into the target cell. By contrast, in contact-dependent inhibition (CDI) systems, a conformational change in a single protein on the surface of the producer cell triggers the delivery of a toxin through the target cell’s OM; however, many of the molecular details of that process remain unknown.

CDI systems are widespread in gram-negative species and typically consist of three proteins: CdiA, CdiB, and CdiI (10, 11, 12, 13, 14, 15). CdiA and CdiB constitute a two-partner secretion system, in which CdiB, a member of the Omp85 family of proteins, exports CdiA to the cell surface (16, 17, 18). CdiA is a large (∼300 kDa) protein with a complex domain architecture that is assembled in a directional (N- to C-terminal) manner (Fig. 1, *A* and *B*). CdiA’s FHA-1 domain forms a β-helical filament that extends approximately 33 nm from the surface of the producer cell (*i.e.*, the inhibitor cell), and it has a receptor-binding domain (RBD) at its distal end (19). Different CdiA homologs bind to different target cell receptors, which include the outer membrane proteins (OMPs) BamA, OmpC/F, and Tsx and the OM lipid lipopolysaccharide (20, 21, 22, 23). The CdiA RBDs can distinguish between homologs of the same receptor in different species and thereby allow for highly selective killing (24).

**Figure 1:**
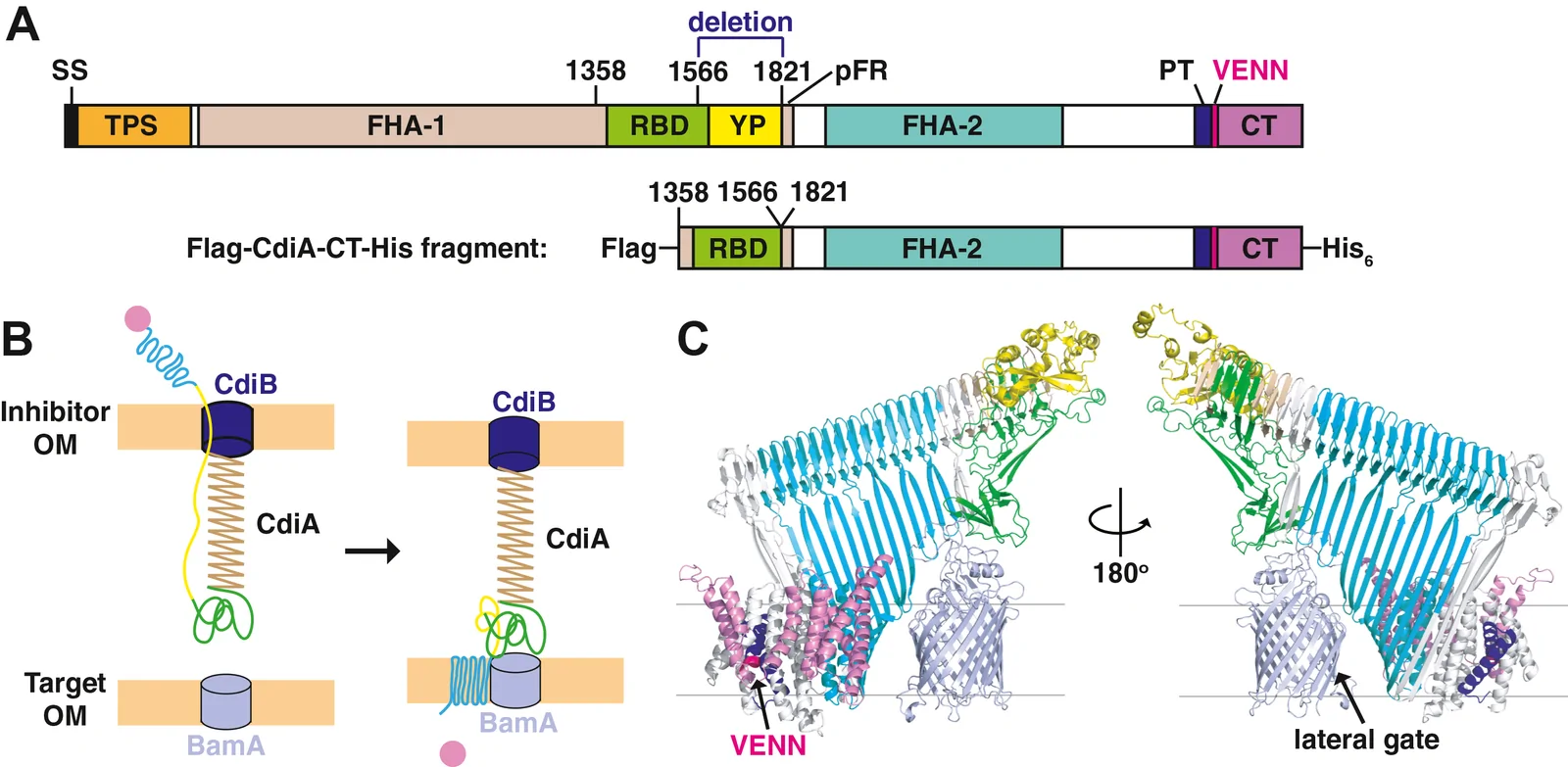
**Overview of CdiA structure and the toxin delivery process.***A*, domain architecture of the full-length *E. coli* EC93 CdiA protein and the functional fragment used in this study. CdiA is directed to the periplasm of the producer cell by an N-terminal signal sequence (SS) and then to CdiB in the OM by a two-partner secretion (TPS) domain. The FHA-1 domain forms a filament on the surface of the cell and is followed by the receptor-binding domain (RBD), the YP domain, and the FHA-2 domain. The pretoxin (PT) domain precedes the conserved VENN cleavage site sequence and the C-terminal toxin (CT) domain. *B*, cartoon of the current model of the contact-dependent inhibition process, in which association of the CdiA^EC93^ RBD with its BamA receptor on the surface of a target cell induces a conformational change that results in the assembly of the FHA-2 domain into the target cell OM and the autoproteolytic release of the CT into the target cell periplasm. The CdiA domains are colored as in *A*. *C*, AlphaFold2 multimer model of CdiA (amino acids R1358-K3132) with the BamA β-barrel (amino acids N422-W810). BamA is colored *light blue,* and the domains of CdiA are colored as in A. Horizontal *gray lines* depict the approximate boundaries of the OM lipid bilayer. OM, outer membrane.

CdiA’s YP region, which follows the RBD, initially adopts an extended conformation that causes CdiA to form a hairpin structure in which the FHA-2, pretoxin, and C-terminal toxin (CT) domains are retained within the inhibitor cell’s periplasm (Fig. 1*B*) (19). Part of the YP domain has consequently been termed the secretion arrest domain (25), but once the RBD binds to a receptor in the OM of a target cell, the arrest is overcome and secretion of the C-terminal domains continues (19). The FHA-2 domain then associates with the OM of the target cell, and the CT domain is released into the target cell’s periplasm by autoproteolysis (19, 26). This autoproteolysis occurs through cyclization of the final asparagine in a conserved cleavage site sequence, which is VENN in most Enterobacteriaceae CdiA homologs (11). Once inside the target cell, the CT may be inserted into the IM or transported across it, depending on its structure and function (as an ionophore or a nuclease) (2, 15, 27). The final component of CDI systems, CdiI, is an immunity protein that inhibits the activity of the toxin inside the producer cell to protect that cell (and its siblings) from death (11, 15).

Although the main steps in the CDI process have been identified, it remains unclear how the toxin translocates through the target OM and what role, if any, the receptor plays in that process. The OM is a dense, protein-rich membrane that contains lipopolysaccharides in its outer leaflet; it acts as a permeability barrier that excludes many small molecules with molecular weights greater than 600 Da (28, 29, 30). It is therefore surprising that CdiA can efficiently deliver its CT domain through this membrane. In this study, we have focused on one CdiA-receptor pair to better understand the toxin translocation mechanism. The CdiA homolog from *Escherichia coli* strain EC93 utilizes BamA as its receptor in the OMs of other *E. coli* (20, 24, 31). BamA is the central component of the five-protein BamABCDE complex, which assembles β-barrel proteins in the OMs of all gram-negative bacteria (32, 33, 34, 35). BamA assembles these OMP substrates using a flexible lateral gate in its own β-barrel, and this unusual feature in BamA’s structure also induces disorder in the surrounding lipid bilayer (36, 37, 38). These features of BamA could potentially also facilitate CdiA toxin translocation.

An AlphaFold2 model of the C-terminal region of CdiA^EC93^ (amino acids R1358-K3132) with BamA’s β-barrel domain provides some insight into how CdiA might interact with a target’s OM (Figs. 1*C* and S1). In agreement with previous experiments, the structure suggests that CdiA’s RBD binds to BamA’s extracellular loops (20, 24, 31). The FHA-2 domain is predicted to form a large β-sheet that extends from a β-helical filament into the OM lipid bilayer adjacent to the lateral gate in BamA’s β-barrel. The stability provided by the extensive network of interstrand hydrogen bonds in this FHA-2 structure might be sufficient to drive the spontaneous insertion of the β-sheet into the membrane. However, CdiA’s pretoxin and CT domains are predicted to form α-helical structures, and their exact locations are more variable in the AlphaFold2 models (Fig. S1). It is not obvious from these models what drives the translocation of the CT domain *through* the OM. Since the CdiA structure assembles from N to C terminus (as it is exported from CdiB), the FHA-2 domain would be expected to fold before the pretoxin and CT domains. What drives the continued movement of the CT into the target periplasm? To gain insight into this process, we developed an *in vitro* assay to monitor the autoproteolytic cleavage of the CT domain from CdiA^EC93^ as the protein assembles into lipid vesicles containing the BamA receptor. This assay allows us to evaluate BamA’s role in the process and to identify steps in the toxin delivery mechanism.

## Results

### CdiA fragment spontaneously cleaves its toxin domain in proteoliposomes containing BamA

The C-terminal half of CdiA contains the domains that are responsible for delivering the toxin through the OM of a target cell. We therefore purified a large CdiA fragment consisting of amino acids R1358-K3132 of the full-length protein but with an internal deletion of residues P1567-N1820 and with an N-terminal Flag-tag and a C-terminal hexahistidine-tag (Fig. 1*A*). This CdiA construct contains the RBD, the FHA-2 domain, and the pretoxin and CT domains. The internal deletion removes the YP domain because constructs containing it were not well expressed and previous studies demonstrated that deletions in this region did not abrogate CDI in cells grown on solid media (19). The resulting Flag-CdiA(R1358-K3132 ΔP1567-N1820)-His construct (which is referred to as Flag-CdiA-CT-His hereafter) is similar to a construct we used previously to characterize the autoproteolytic mechanism by which CdiA cleaves itself to release its C-terminal domain (26). Those previous experiments established the functionality of the purified CdiA fragment, but they were carried out in detergent solution and consequently did not examine how CdiA interacts with its receptor in a membrane. To study these membrane-associated steps in the CDI process, we developed methods to monitor the cleavage of the CT domain from the Flag-CdiA-CT-His fragment in BamA-containing proteoliposomes.

The Flag-CdiA-CT-His protein was purified in an unfolded state in urea solution, and BamA and the complete BamABCDE complex were purified in detergent solution and then incorporated into proteoliposomes composed of 1,2-dimyristoyl-sn-glycero-3-phosphocholine (DMPC) phospholipids. We first verified that the proteoliposomes contain approximately equal amounts of the Bam proteins by SDS-PAGE and determined that a substantial percentage of the BamA proteins are properly oriented in the membranes to interact with CdiA (*i.e.*, with their N-terminal POTRA domains in the lumen of the vesicles) by a protease protection assay (Fig. S2). We then added the Flag-CdiA-CT-His protein to the BamA and BamABCDE proteoliposomes and to “empty” liposomes that were prepared with the same lipids but without any Bam proteins. These reaction mixtures were incubated at 25 °C for 4 h, and the samples were then analyzed by SDS-PAGE and immunoblotting with anti-Flag and anti-His antibodies to determine whether the Flag-CdiA-CT-His protein cut itself into two pieces, releasing the CT domain (Fig. 2*A*). Indeed, two additional bands appear in the samples containing the BamA and BamABCDE proteoliposomes after 4 h: a Flag-tagged species with a molecular weight of approximately 134 kDa and a His-tagged species with a molecular weight of approximately 24 kDa (Fig. 2*A*). These molecular weights and epitope tags are consistent with the expected autoproteolysis occurring after the final asparagine (N2908) of the conserved VENN cleavage sequence.

**Figure 2:**
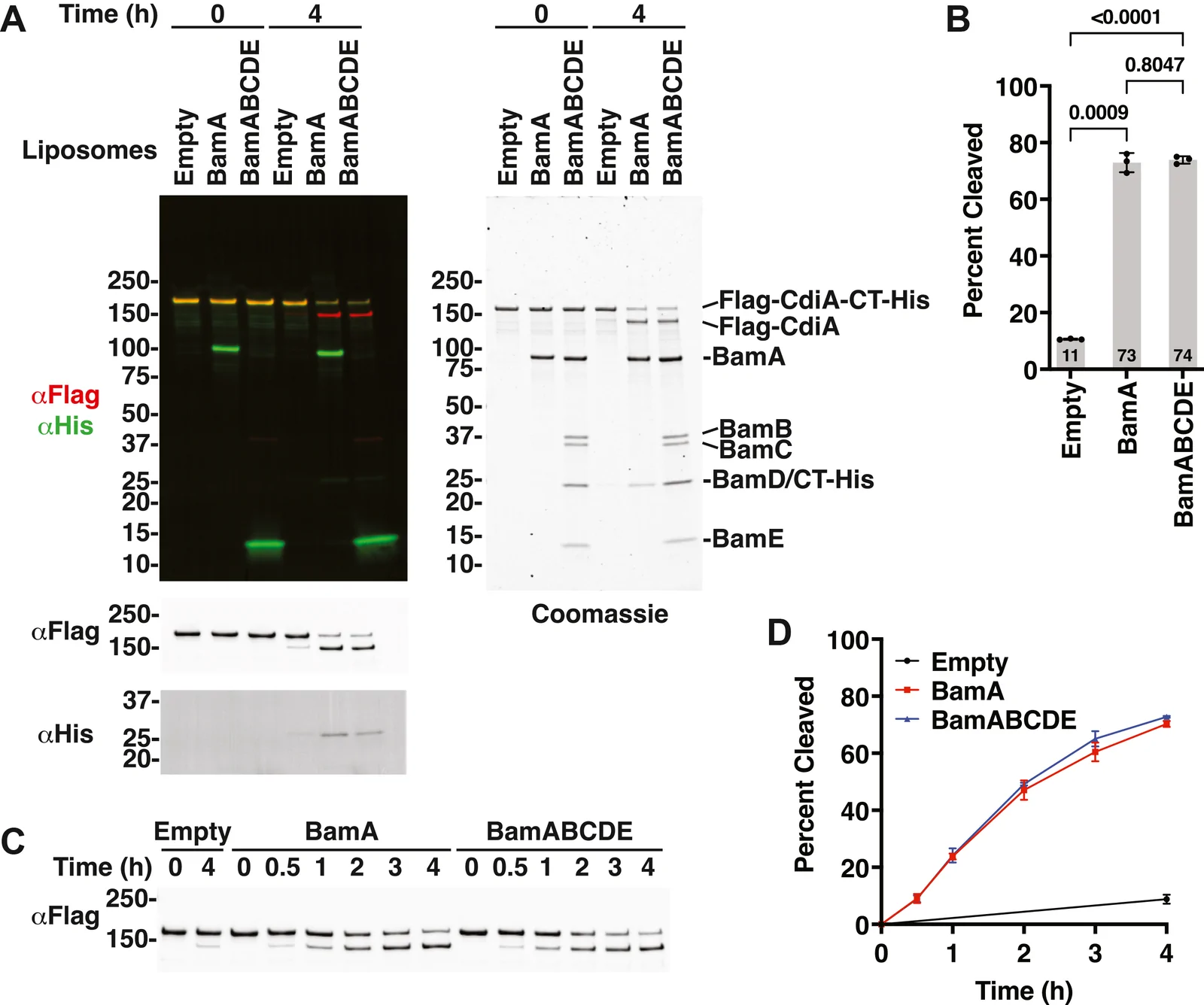
**CdiA fragment efficiently cleaves its CT domain in proteoliposomes containing BamA.***A*, immunoblot (*left*) and Coomassie-stained SDS-PAGE (*right*) analysis of Flag-CdiA-CT-His autoproteolytic cleavage in empty liposomes and liposomes containing purified His-BamA or BamABCDE-His complexes. The final concentrations of the Flag-CdiA-CT-His and Bam proteins in the reaction mixtures were 1 μM and 2.5 μM, respectively. Samples were collected immediately after addition of the Flag-CdiA-CT-His fragment to the liposomes and after 4 h of incubation at 25 °C. Flag-tagged species were detected with anti-Flag DyLight 680 antibodies (*red*, *upper left panel*), and His-tagged species were detected with anti-His CF 488A antibodies (*green*, *upper left panel*). The uncleaved protein appears *yellow* due to the presence of both tags. Cleavage efficiency was quantified by comparing the intensities of the uncleaved (Flag-CdiA-CT-His) and cleaved (Flag-CdiA) bands in the anti-Flag immunoblot (*grayscale*, *middle left panel*). The cleaved CT-His fragment is detected after 4 h in the anti-His immunoblot (*grayscale*, *bottom left panel*) and in the Coomassie-stained gel at the same molecular weight as BamD (*right panel*). *B*, the Bam lipoproteins do not significantly affect the efficiency of CdiA cleavage in proteoliposomes. The percentage of cleaved CdiA after 4 h of incubation was quantified based on the relative intensities of the Flag-tagged bands in three independent replicates of the experiment in *A*. The mean values are depicted, and the error bars represent the standard deviation among the replicates. *p*-values for the indicated pairwise comparisons were calculated by a one-way ANOVA test followed by a Tukey’s multiple comparisons test. *C*, the rates of CdiA cleavage in BamA and BamABCDE proteoliposomes are indistinguishable. The Flag-CdiA-CT-His fragment was incubated with empty liposomes and proteoliposomes as in *A*. Samples were removed at the indicated time points, quenched, and then analyzed by immunoblotting. *D*, the percentage of cleaved CdiA at each time point was quantified based on the relative intensities of the Flag-tagged bands in three replicates of the experiment in *C*. Error bars represent the standard deviation from the mean at each time point. CT, C-terminal toxin.

We quantified the efficiency of autoproteolytic cleavage in each sample by comparing the intensities of the cleaved and uncleaved bands in the anti-Flag immunoblot at the endpoint of the experiment (Fig. 2*B*) and at time points throughout the incubation period (Fig. 2, *C* and *D*). The rates of Flag-CdiA-CT-His cleavage in the BamA and BamABCDE proteoliposomes were indistinguishable, resulting in more than 70% cleaved protein in 4 h. Significantly less cleaved protein (11%) was produced in the empty liposomes over the same time period. This simple proteoliposome system thus recapitulates the receptor-dependent CdiA toxin cleavage process that is observed *in vivo*. Moreover, our data are consistent with genetic experiments that demonstrated that the Bam lipoproteins (BamB, C, D, and E) are not required for CDI *in vivo* (20).

It is worth noting that *E. coli* membranes do not contain phosphatidylcholine (PC) lipids, and we initially tested different types of phospholipids in our assay, including several that have been used to study the assembly of OMPs (Fig. S3) (39, 40, 41, 42, 43, 44, 45, 46). Proteoliposomes prepared from an extract of polar *E. coli* phospholipids or from short chain phosphatidylglycerol or phosphatidylethanolamine lipids did not allow for CdiA toxin cleavage. We only observed toxin cleavage in proteoliposomes prepared with PC lipids containing saturated fatty acids of 10, 12, or 14 carbons—with increasing efficiency with increasing fatty acid length in that range. PC lipids with longer saturated fatty acids (16 or 18 carbons) or with unsaturated fatty acids did not allow for toxin cleavage. Collectively, these observations suggest that the lipid head groups and the hydrophobic thickness of the bilayer influence the efficiency of the CDI process. It is not surprising that CdiA is sensitive to the lipid composition of the proteoliposomes because many studies have demonstrated that different lipids can significantly alter the rates at which different OMPs assemble (40, 42, 43, 44, 45, 46). We also previously observed that the efficiency of CdiA cleavage in detergent solution was highly dependent on the detergent’s alkyl chain length (26). Perhaps CdiA assembles into DMPC lipids most efficiently because the hydrophobic thickness of DMPC bilayers (24–25 Å) is comparable to that of the *E. coli* OM (47, 48). We carried out all our subsequent experiments in DMPC proteoliposomes, and the CdiA toxin cleavage process in this system recapitulates many aspects of the *in vivo* process. It remains an open question, however, how CdiA interacts with the lipopolysaccharides and phospholipids that compose the OM.

We next analyzed the effects of amino acid substitutions in both the Flag-CdiA-CT-His fragment and BamA to confirm that these proteins specifically interact in the proteoliposomes and that the toxin cleavage occurs at the expected site. An N2908A substitution has been shown to eliminate toxin cleavage *in vitro* and *in vivo* (19, 26), and we first confirmed that a Flag-CdiA-CT-His protein containing this substitution does not undergo cleavage in proteoliposomes with or without BamA (Figs. 3*A* and S4*A*). We then analyzed the effects of substitutions that reduce the affinity of the BamA-CdiA interaction. In the course of other experiments in our laboratory, we identified a single amino acid substitution in the CdiA RBD, W1519A, that reduces the affinity of its interaction with BamA. Similarly, we found that substitutions at three positions in BamA’s extracellular loop 4 (Y550N/E554Q/H555Y) reduce the receptor’s interaction with the wildtype CdiA RBD (Fig. S4, *B* and *C*). The RBD interacts with this part of loop 4, and the E554Q and H555Y substitutions were previously shown to provide resistance to a bactericidal, monoclonal antibody that binds to the same region of BamA (31, 49). We found that these substitutions significantly reduce the efficiency of Flag-CdiA-CT-His toxin cleavage in our proteoliposome assay (Figs. 3, *A*, *B*, and S4*A*).

**Figure 3:**
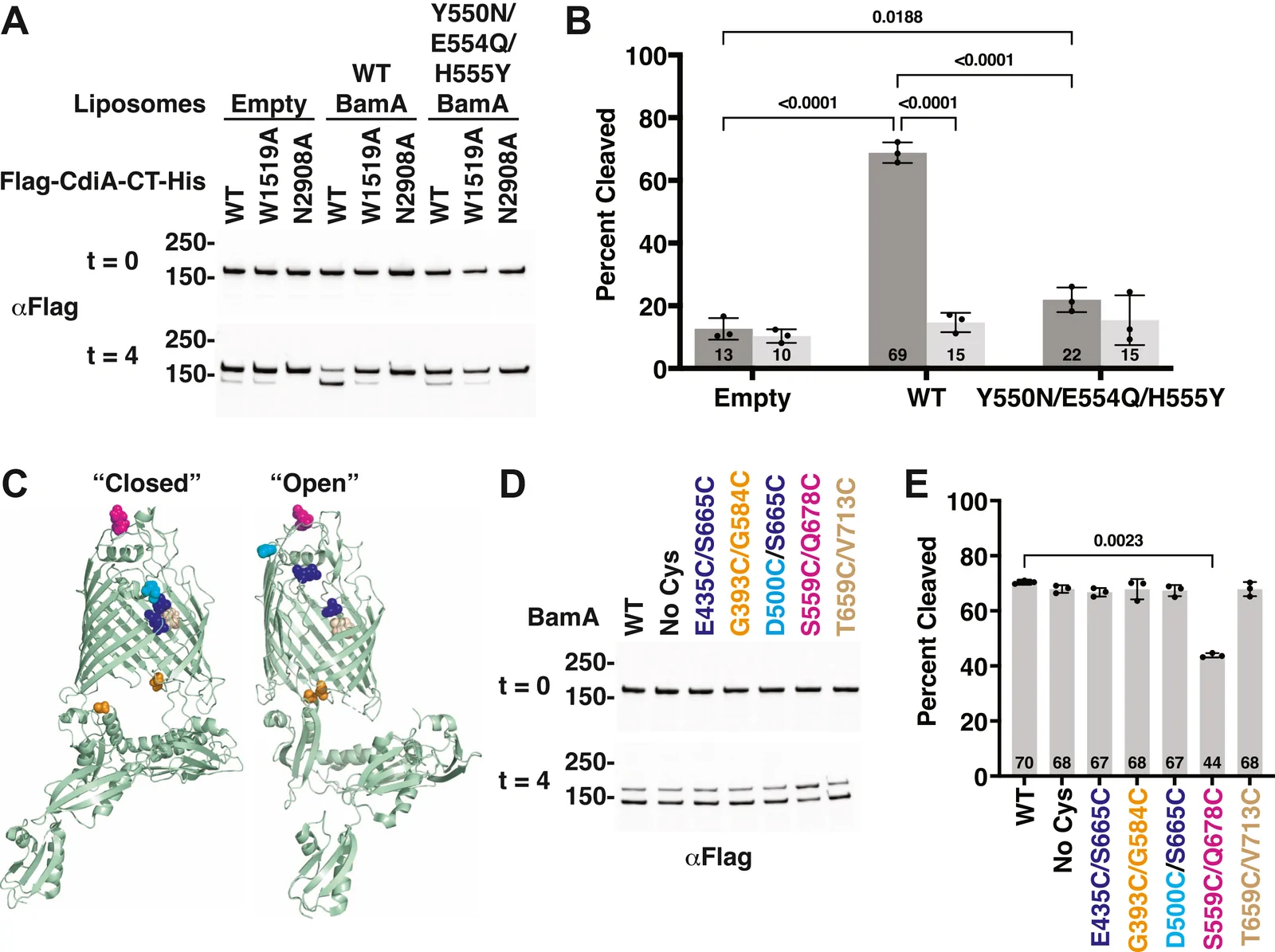
**CdiA toxin cleavage in proteoliposomes depends on specific interactions with BamA.***A*, amino acid substitutions in CdiA’s RBD and in BamA’s extracellular loop 4 reduce the efficiency of toxin cleavage in proteoliposomes. Wildtype and altered forms of the Flag-CdiA-CT-His fragment were incubated for 4 h at 25 °C with empty liposomes and with proteoliposomes containing either wildtype BamA or Y550N/E554Q/H555Y BamA. The W1519A substitution is in CdiA’s RBD, and the N2908A substitution prevents toxin cleavage by altering the final asparagine of the VENN cleavage sequence. *B*, quantification of the cleavage efficiencies of the wildtype (*dark gray*) and W1519A (*light gray*) CdiA fragments in three replicates of the experiment in *A*. The mean values are depicted, and the error bars represent the standard deviation among the replicates. *p*-values for the indicated pairwise comparisons were calculated by a two-way ANOVA test followed by a Tukey’s multiple comparisons test. All other pairwise comparisons were not statistically significant (*p* > 0.05). *C*, locations of the pairs of amino acids that were substituted with cysteines to generate disulfide-bonded forms of BamA. The altered amino acids are displayed in space-filling rendering and colored as in *D* and *E*; their positions are depicted on BamA structures in the closed (*left*, pdb: 5d0o) and open (*right*, pdb: 5d0q) conformations. *D*, CdiA toxin cleavage is impeded by a disulfide crosslink between extracellular loops 4 and 6 in BamA but not by crosslinks that constrain BamA’s lateral gate. The wildtype Flag-CdiA-CT-His fragment was incubated with proteoliposomes containing the indicated forms of BamA in their oxidized, disulfide-bonded states for 4 h at 25 °C. The native cysteines in BamA (C690 and C700) were substituted with serines in the “No Cys” form and in all the forms containing non-native cysteines. *E*, quantification of three replicates of the experiment in *D* demonstrates that only the 559 to 678 disulfide bond in BamA results in a statistically significant reduction in the CdiA cleavage efficiency. The mean cleavage percentages after 4 h are indicated, and the error bars represent the standard deviation among the replicates. *p*-values were calculated by a one-way ANOVA test followed by a Dunnett’s multiple comparisons test. The *p*-values of the pairwise comparisons that were not significant (*p* > 0.05) are not depicted. RBD, receptor-binding domain.

The cleavage efficiency of W1519A Flag-CdiA-CT-His in BamA proteoliposomes is not significantly different from that observed in the empty liposomes, suggesting that the W1519A protein undergoes autoproteolysis through a less efficient, BamA-independent mechanism. The Y550N/E554Q/H555Y substitution in BamA also significantly reduces the amount of cleaved CdiA observed after 4 h, but the effect is not as potent as that of the W1519A substitution in CdiA; slightly more WT Flag-CdiA-CT-His undergoes proteolytic cleavage in the Y550N/E554Q/H555Y BamA proteoliposomes than in the empty liposomes. Nevertheless, these experiments demonstrate that the *in vitro* CDI toxin cleavage process requires the formation of specific interactions between the CdiA RBD and BamA’s extracellular surface (19, 22, 24).

### BamA’s lateral gating mechanism is not required for CdiA toxin cleavage

Numerous structural studies have established that the lateral gate in BamA’s β-barrel is highly plastic (50, 51, 52, 53, 54, 55). The N- and C-terminal strands of the barrel can hydrogen bond to one another in what has been termed the “closed” or “inward open” state, but the N-terminal strand of BamA’s barrel can also hydrogen bond to OMP substrates. The first several strands of BamA’s barrel flex outward to various “open” or “outward open” conformations as an OMP substrate assembles into the membrane. Given that CdiA’s FHA-2 domain is predicted to form an extended β-sheet structure, we considered the possibility that BamA’s lateral gate might assist in assembling the FHA-2 domain in a manner akin to how it assembles OMPs. We therefore tested the effect of several intramolecular disulfide bonds in BamA, which restrict the conformational flexibility of different parts of its structure.

We prepared proteoliposomes containing five different disulfide-bonded variants of BamA. In each of these variants, BamA’s two native cysteines in extracellular loop 6 (C690 and C700) were substituted with serines, and two new cysteines were introduced at other positions (Fig. 3*C*). The E435C/S665C and D500C/S665C variants contain disulfide bonds that are expected to stabilize the “closed” state of the β-barrel because they link extracellular loop 1 to loop 6 or loop 3 to loop 6, respectively. The G393C/G584C disulfide bond links POTRA domain 5 to periplasmic turn 4 of the barrel, and this variant has been used as a proxy for the “open” state of the barrel (52, 56, 57). Some recent studies have indicated that the lateral gate retains some flexibility even when the E435C/S665C and G393C/G584C disulfide bonds are formed such that these variant proteins may not be completely “closed” or “open”; however, both disulfide linkages reduce the efficiency of β-barrel assembly *in vitro* (52, 58). We also prepared an S559C/Q678C BamA, which contains a linkage between extracellular loops 4 and 6, and a T659C/V713C BamA, which contains a linkage between a region of loop 6 that is folded into the lumen of the β-barrel and the interior wall of the β-barrel (59).

Only the disulfide bond between loop 4 and loop 6 (in the S559C/Q678C variant) reduces the amount of cleaved CdiA toxin in the proteoliposomes after 4 h of incubation; the other disulfide-bonded variants all behave like wildtype BamA (Figs. 3, *D*, *E*, and S5*A*). CdiA’s RBD has previously been shown to interact with loops 4 and 6 of BamA, and AlphaFold2 predicts that both the RBD and FHA-2 domain are in close proximity to this region of BamA (Fig. 1*C*) (24, 31). We therefore performed a simple copurification experiment to determine whether the 559 to 678 disulfide bond disrupts the interaction with the CdiA RBD (Fig. S5*B*). The RBD copurified with wildtype BamA, but not with the S559C/Q678C disulfide-bonded variant; the reduction in toxin cleavage is therefore likely due to a weaker initial interaction between the Flag-CdiA-CT-His fragment and the disulfide-bonded BamA. We conclude from these experiments that although CdiA binds near BamA’s lateral gate, the CdiA toxin cleavage process does not require BamA’s ability to open and close, and it is unlikely that CdiA interacts with BamA’s lateral gate in the same manner as OMP substrates.

### CdiA rapidly becomes membrane-associated but slowly cleaves its toxin

Cellular experiments have indicated that when CdiA delivers its toxin into a target cell, its FHA-2 domain forms a stable association with that cell’s OM (19). We therefore performed a sedimentation experiment to examine whether the Flag-CdiA-CT-His fragment inserts into the proteoliposomes in our assay system. The wildtype Flag-CdiA-CT-His was added to proteoliposomes containing either wildtype BamA or Y550N/E554Q/H555Y BamA. After an incubation period of 30 min or 4 h, these reaction mixtures were centrifuged to separate the proteoliposomes from proteins in the supernatant. The pellets after this initial centrifugation were then washed with a sodium carbonate solution, which has been shown to remove proteins that are weakly or peripherally associated with membranes (60), and the samples were then centrifuged again (Fig. 4*A*). The amount of CdiA in the final postwash pellet was compared to the amount in the original, precentrifugation sample to determine what percentage was stably associated with the proteoliposomes (Fig. 4*B*). (To account for any differences in how well the proteoliposomes sedimented in different experiments, the amount of CdiA in each sample was first normalized to the amount of BamA in the same sample such that the ratio of CdiA: BamA in each precentrifugation sample was compared to the ratio in the post-wash pellet.) Surprisingly, the majority (79%) of the CdiA protein sediments with the wildtype BamA proteoliposomes after just 30 min of incubation time, and a longer incubation period does not increase the amount of CdiA in the final pellet. In fact, a slightly smaller amount of CdiA was found in the pellet after 4 h, but the difference was not statistically significant. By contrast, significantly less CdiA sedimented with the Y550N/E554Q/H555Y BamA proteoliposomes than with the wildtype BamA proteoliposomes at both time points. The Flag-CdiA-CT-His protein is not, therefore, simply precipitating in a nonspecific manner; the amount of CdiA in the pellet correlates with the strength of its interaction with the BamA in the proteoliposomes. The resistance of the CdiA protein to removal from the pellet by the sodium carbonate solution also suggests that it is embedded in the membranes.

**Figure 4:**
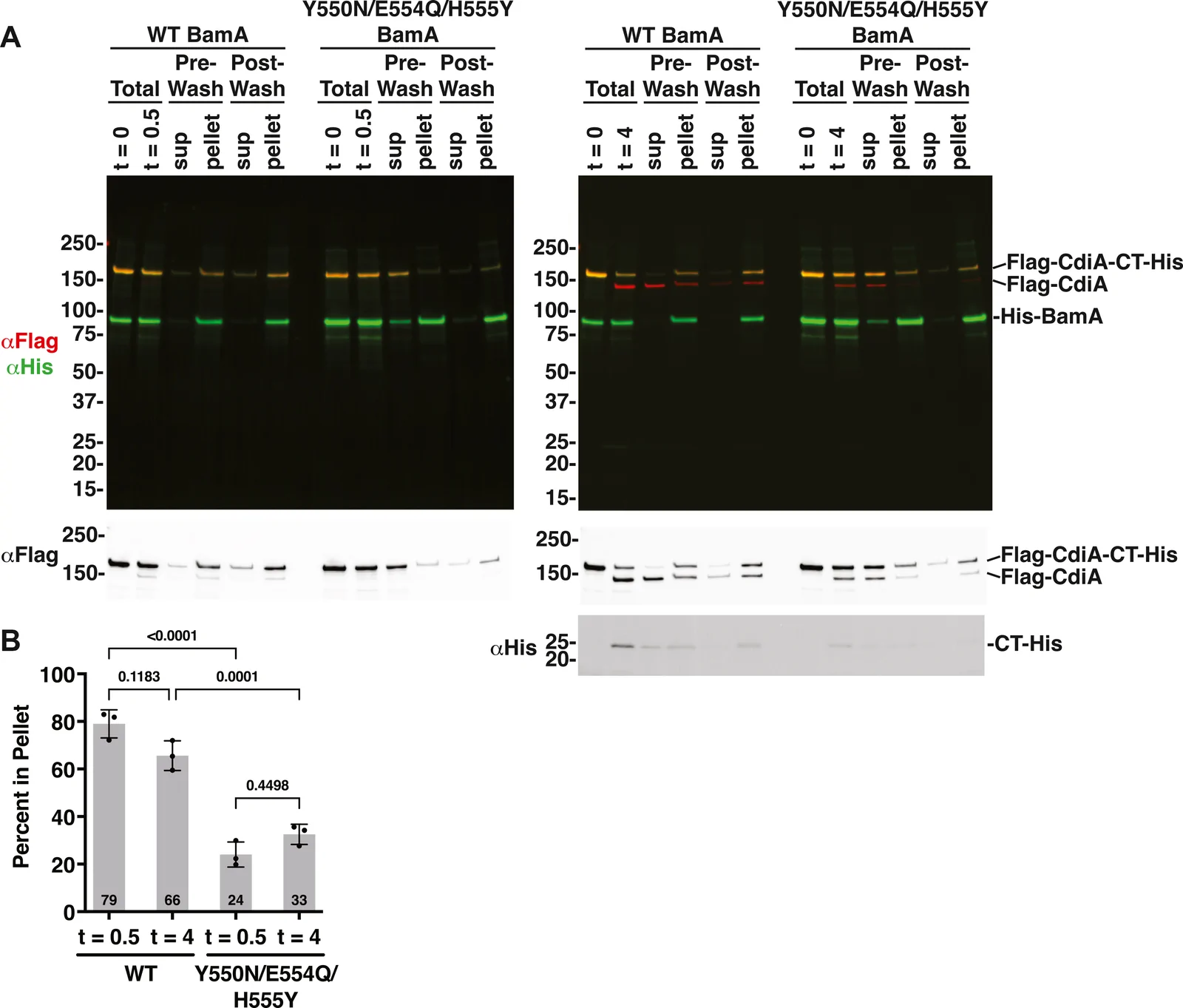
**CdiA rapidly inserts into proteoliposomes containing wildtype BamA but slowly cleaves its CT domain.***A*, wildtype Flag-CdiA-CT-His was incubated with proteoliposomes containing either wildtype or Y550N/E554Q/H555Y BamA for 0.5 h (*left panels*) or for 4 h (*right panels*). The samples were subsequently centrifuged, and the pellet fractions were washed with a sodium carbonate solution and centrifuged again. Samples of the precentrifugation reactions and of the prewash and postwash supernatant (sup) and pellet fractions were analyzed by immunoblotting. *B*, significantly less CdiA inserts into proteoliposomes containing the Y550N/E554Q/H555Y BamA than into those containing wildtype BamA, but the amount of membrane-inserted CdiA at t = 4 h is not significantly different from that at t = 0.5 h. The amount of membrane-inserted CdiA in the experiment in *A* was determined by comparing the ratio of Flag-tagged CdiA to His-tagged BamA in the postwash pellet fractions to the ratio present in the total (precentrifugation) samples. The mean values from three replicate experiments are displayed, and the error bars represent the standard deviation from the mean. *p*-values for the indicated pairwise comparisons were calculated by a two-way ANOVA test followed by a Tukey’s multiple comparisons test. CT, C-terminal toxin.

Although the amount of CdiA in the proteoliposomes does not change significantly between 30 min and 4 h, the autoproteolytic cleavage of the protein continues to occur throughout this period. The toxin cleavage step is therefore the slow step in the mechanism *in vitro*. We detected the cleaved toxin in the wildtype BamA proteoliposome pellet after the 4-h reaction period and found that it was resistant to carbonate extraction (Fig. 4*A*, bottom right panel). The toxin thus seems to remain in the proteoliposome membranes after it is autoproteolytically cleaved. In a target cell, the CdiA^EC93^ toxin is predicted to form an ionophore in the cell’s IM, which dissipates the proton motive force across that membrane (27, 61). The toxin is therefore expected to be an integral membrane protein, but *in vivo,* it must be transported across the periplasm to its final destination in the IM. We would not expect our simple assay system to recapitulate these subsequent IM-associated steps in the CDI process, but we considered the possibility that additional factors might be required to transport the toxin through the OM and across the target cell’s periplasm.

### OMP chaperones facilitate CdiA^EC93^ toxin cleavage

The periplasm contains a network of chaperones that assist in the transport and assembly of proteins in the cell envelope (62, 63). Among these, SurA is the primary chaperone for most OMPs that are assembled by the Bam complex (64, 65, 66, 67, 68). SurA binds unfolded OMPs in the periplasm after they are secreted across the IM and delivers them to the Bam complex in the OM by interacting with BamA’s periplasmic POTRA domains and BamB (69, 70). Cells lacking SurA were also previously shown to be mildly resistant to the EC93 CDI system (20). This resistance was attributed to an upregulation of the colanic acid biosynthesis pathway because inactivating colanic acid production in the *surA* mutant reversed its resistance. Colanic acid capsule production independently confers CDI resistance—most likely by obstructing the initial CdiA–BamA interaction on the target cell’s extracellular surface (20). We nevertheless considered the possibility that SurA might also directly participate in CDI at a later stage in the process by binding the toxin as it is released into the target cell’s periplasm.

In order to incorporate SurA efficiently into the lumen of the proteoliposomes in our assay, we prepared disulfide-bonded Bam–SurA complexes using a previously reported pair of engineered cysteines in the first POTRA domain of BamA (R76C) and the core domain of SurA (K27C) (Fig. S2) (69). The rate of Flag-CdiA-CT-His toxin cleavage in proteoliposomes containing these complexes was faster than in those containing only the Bam complex by a small, but reproducible, amount (Figs. 5*A* and S6*A*). The rate of cleavage was approximately 1.5 times faster in the SurA-containing proteoliposomes (Fig. S6*B*). Notably, the toxin cleavage rate was not significantly faster when SurA was added externally to proteoliposomes prepared with only the Bam complex or when SurA was incorporated inside the Bam complex proteoliposomes by including it in the buffer used to prepare them (Figs. 5*A* and S6, *A*, *B*). These results imply that SurA increases the rate of toxin cleavage when it is bound to the Bam complex in the proteoliposomes, but not when it is free in solution. SurA is not simply acting as a generic chaperone that increases the cleavage rate by maintaining the solubility or foldability of the CdiA protein before it binds to BamA; it exerts its effect in concert with the Bam complex.

**Figure 5:**
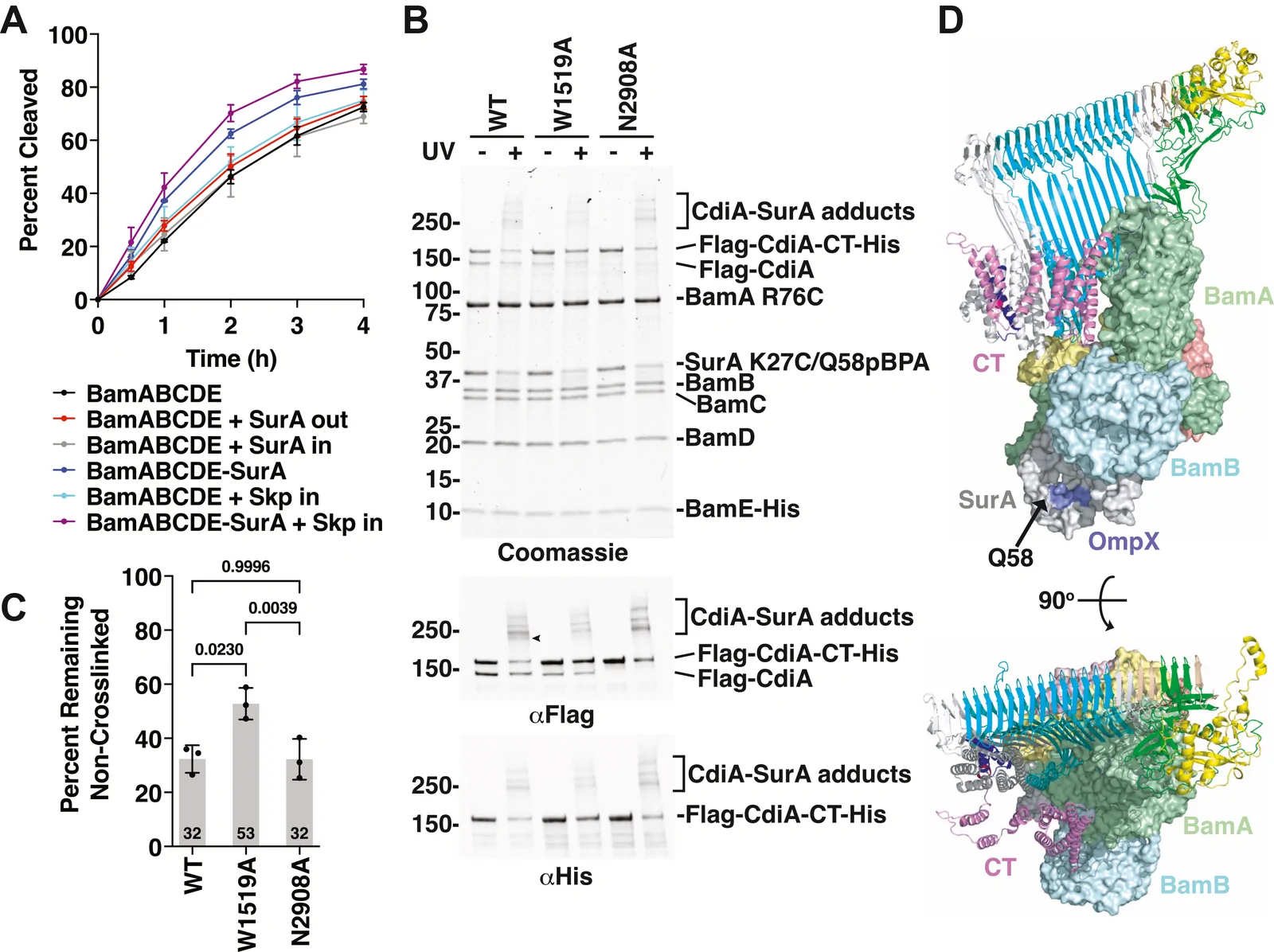
**SurA and Skp increase the rate of toxin cleavage *in vitro.****A*, time course of Flag-CdiA-CT-His cleavage in proteoliposomes containing BamABCDE complexes with and without chaperone proteins. SurA was added externally to proteoliposomes containing BamABCDE complexes (BamABCDE + SurA out, *red*), incorporated into BamABCDE proteoliposomes during their preparation (BamABCDE + SurA in, *gray*), or attached to BamA in disulfide-bonded BamA(R76C)BCDE-SurA(K27C) complexes in the proteoliposomes (BamABCDE-SurA, *dark blue*). Skp was incorporated into proteoliposomes containing BamABCDE or BamA(R76C)BCDE-SurA(K27C) complexes during their preparation (BamABCDE + Skp in, *cyan*; BamABCDE-SurA + Skp in, *purple*). The wildtype Flag-CdiA-CT-His fragment was incubated with the proteoliposomes at 25 °C. Samples were removed and quenched at the indicated time points and then analyzed by SDS-PAGE and immunoblotting with anti-Flag DyLight 680 and anti-His CF 488A antibodies. The cleavage percentages were determined by comparing the intensities of the Flag-CdiA-CT-His (uncleaved) and Flag-CdiA (cleaved) bands at each time point in three replicate experiments. Error bars represent the standard deviation from the mean. *B*, SurA’s N-terminal core domain photo-crosslinks to CdiA in proteoliposomes. Wildtype and altered forms of the Flag-CdiA-CT-His fragment were added to proteoliposomes containing disulfide-bonded BamA(R76C)BCDE-SurA(K27C/Q58*p*BPA) complexes. After 1 h of incubation at 25 °C, samples were irradiated with UV light for 20 min to induce photocrosslinking. The irradiated (+UV) and unirradiated (-UV) samples were then analyzed by SDS-PAGE under reducing conditions and stained with Coomassie Blue (*top panel*) or immunoblotted with anti-Flag DyLight 680 and anti-His CF 488 antibodies (*middle* and *bottom panels*). Crosslinked CdiA-SurA adducts were identified as high molecular weight Flag-tagged species in the immunoblot. An adduct that appears in the anti-Flag blot but not in the anti-His blot is indicated by a *black arrowhead*. *C*, CdiA-SurA crosslinking efficiency correlates with CdiA binding to BamA. The W1519A Flag-CdiA-CT-His crosslinks less efficiently to SurA than the wildtype and noncleavable (N2908A) forms of Flag-CdiA-CT-His. The percentage of CdiA remaining noncrosslinked after UV irradiation was determined by comparing the total intensities of the Flag-CdiA-CT-His (uncleaved) and Flag-CdiA (cleaved) bands in the -UV and +UV samples. The depicted data represent the average of three replicates of the experiment in *B*. Error bars depict the standard deviation, and *p*-values for the indicated pairwise comparisons were calculated by a one-way ANOVA test followed by a Tukey’s multiple comparisons test. *D*, AlphaFold2 multimer model of CdiA-BamA overlaid with a cryo-EM structure of a BamABCDE-SurA-OmpX complex (pdb: 8qpw). The CdiA-BamA structure is shown in cartoon representation, and the BamABCDE-SurA-OmpX complex is shown in surface representation. The CdiA CT domain (*lilac*) is positioned in the membrane above SurA (*gray*), suggesting SurA could bind the CT if it adopts an extended conformation. The location of amino acid Q58 in SurA is indicated by the *black arrow* adjacent to the OmpX fragment (*lavender*). CT, C-terminal toxin.

The Bam–SurA interaction is relatively weak with a reported K_d_ of 2.6 μM (71). Both the Bam complex and SurA were present at concentrations of approximately 2.5 μM in our experiments. Therefore, in the experiments in which SurA is not tethered to Bam, the proteoliposomes likely contain a mixture of SurA-bound and SurA-free Bam complexes. By contrast, in the experiments with the disulfide-bonded Bam–SurA complexes, all of the Bam complexes have a bound SurA molecule. The disulfide tether increases the effective concentration of SurA and ensures that the chaperone is present when CdiA binds to BamA and assembles into the membrane. That increase in effective concentration may explain the faster rate of toxin cleavage in the proteoliposomes containing the disulfide-bonded Bam–SurA complexes, but it is also possible that the disulfide-bonded Bam–SurA complexes adopt a conformation that facilitates CdiA toxin cleavage more effectively than the SurA-free complexes.

We favor the model that SurA directly assists in toxin cleavage because SurA can be photocrosslinked to the Flag-CdiA-CT-His protein in the Bam-SurA proteoliposomes. We prepared disulfide-bonded Bam–SurA complexes that also contain a substitution of amino acid Q58 in SurA with the photoreactive amino acid *p*-benzoylphenylalanine (*p*BPA) (72, 73, 74). We selected residue 58 in the core domain of SurA for substitution because a fragment of an OMP substrate (OmpX) was observed in close proximity to this residue in a cryo-EM structure of the Bam–SurA complex, and we hypothesized that the CdiA toxin might interact with SurA in a manner similar to OMP substrates (69). We incubated proteoliposomes containing the *p*BPA-substituted Bam–SurA complexes with wildtype and mutant forms of the Flag-CdiA-CT-His protein for 1 h and then irradiated the samples with ultraviolet light to activate the *p*BPA to covalently crosslink to nearby proteins. SDS-PAGE and immunoblotting revealed that this site in SurA specifically crosslinks to the CdiA proteins, and not apparently to any of the Bam proteins (Figs. 5*B* and S6*C*). The intensities of the Flag-CdiA-CT-His and SurA bands decrease in the irradiated samples as several Flag-tagged bands of higher molecular weight than the Flag-CdiA-CT-His protein appear. Most of the CdiA-SurA adducts are detected by both anti-Flag and anti-His antibodies, indicating that the toxin domain is still attached to the CdiA protein when it crosslinks to SurA; however, SurA also crosslinks to the Flag-CdiA fragment lacking the toxin domain (see band indicated by arrow in anti-Flag blot in Figs. 5*B* and S6*C*). The core domain of SurA is therefore in close proximity to the CdiA protein in the proteoliposomes.

In several repeated experiments, we noticed that SurA crosslinks to the W1519A Flag-CdiA-CT-His protein less efficiently than to the wildtype and noncleavable N2908A proteins. We quantified this difference by comparing the intensities of the noncrosslinked Flag-CdiA-CT-His (uncleaved) and Flag-CdiA (cleaved) bands in the irradiated and unirradiated samples. Significantly more of the W1519A protein remains noncrosslinked (Fig. 5*C*). The efficiency of CdiA crosslinking to SurA thus correlates with the CdiA-BamA binding affinity. These observations support a model in which the crosslinking to SurA depends on a prior interaction between CdiA and BamA; the CdiA protein first binds to the extracellular surface of BamA, assembles into the proteoliposome membranes, and then is in close proximity to SurA. When the AlphaFold2 model of the BamA–CdiA complex is overlaid with a cryo-EM structure of the Bam–SurA complex with the bound OmpX substrate, the CdiA toxin is positioned above the SurA core domain (Fig. 5*D*). AlphaFold2 predicts that the toxin adopts a folded α-helical structure adjacent to CdiA’s FHA-2 domain in the membrane, but if the toxin instead adopts an unfolded structure that extends into the periplasm, it could plausibly interact with SurA’s core domain—if the AlphaFold2 model is otherwise largely correct.

We also evaluated whether another OMP chaperone, Skp, increases the rate of CdiA toxin cleavage. Skp is an attractive candidate chaperone for transporting the CdiA toxin across the periplasm because it has been shown to remove misfolded OMPs from the Bam complex and to facilitate the insertion of OMPs into the IM under certain circumstances (75, 76). No specific sites of interaction between Skp and the Bam complex have been identified (43, 66), so we incorporated Skp into proteoliposomes containing the Bam complex and the Bam-SurA complex by including it in the buffer used to prepare the proteoliposomes. The rate of Flag-CdiA-CT-His cleavage in the proteoliposomes containing the Bam complex and Skp is slightly, but not significantly higher than in those containing only the Bam complex, but including Skp in the Bam-SurA proteoliposomes increases the cleavage rate (Figs. 5*A* and S6*A*). The rate of toxin cleavage in the proteoliposomes containing the Bam–SurA complex and Skp was approximately 1.8 times faster than the rate in the proteoliposomes containing only the Bam complex (Fig. S6*B*). SurA and Skp may therefore both assist in the toxin cleavage process in some manner.

We analyzed whether SurA and Skp have any effect on the toxin delivery process in cells by culturing inhibitor cells that produce CdiB, CdiA, and CdiI with target cells that overexpress SurA, Skp, or the unrelated periplasmic protein maltose-binding protein (MBP), or that carry an empty vector (Figs. 6 and S7). After 4 h of co-incubation, we analyzed the number of inhibitor and target cells present in each mixture by plating serial dilutions on selective media. (The inhibitor cells are resistant to carbenicillin, and the target cells are resistant to kanamycin.) In several replicate experiments, we found that targets that overexpress SurA are killed more efficiently than those that overexpress MBP or those that carry an empty vector; that is, fewer colony-forming units (cfu) of the SurA-overexpressing cells are present in the cocultures at the end of the experiment. When the target cells are cocultured with inhibitor cells that express the noncleavable N2908A variant of CdiA, SurA overexpression had no discernible effect on the number of remaining cfu when compared to the empty vector and MBP controls. SurA overexpression therefore does not result in a growth defect that would explain the reduced number of target cells in the cocultures with the wildtype inhibitor cells; the reduction depends on CdiA toxin release.

**Figure 6:**
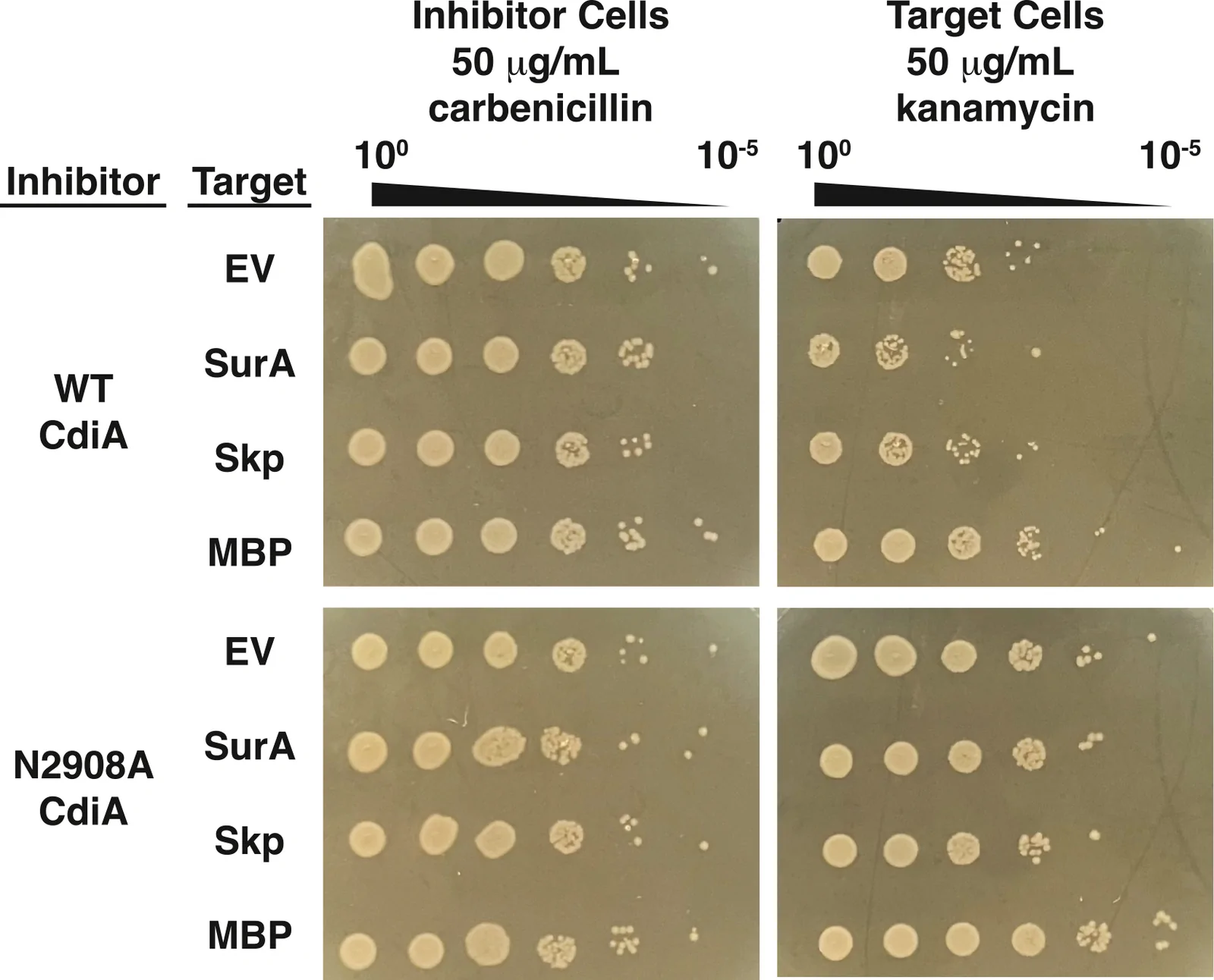
**Target cells that overexpress SurA are more sensitive to killing by CdiA.** Inhibitor cells expressing the *cdiBAI* gene cassette with a wildtype or N2908A version of CdiA were mixed in a 1:1 ratio with target cells overexpressing SurA, Skp, MBP, or an empty vector and cultured in liquid media for 4 h. Samples of the cocultures were serially diluted and plated on selective media to compare the number of colony-forming units of inhibitor (carbencillin-resistant) and target (kanamycin-resistant) cells in each mixture following the incubation period. The displayed results are a representative example from three biological replicates. MBP, maltose-binding protein.

Target cells that overexpress Skp also appear to be killed more efficiently than the control targets in these same experiments; however, fewer Skp-overexpressing cells are present in the cocultures with the control (N2908A) inhibitor cells, so the lower number of cfu may reflect a reduced growth rate. Nevertheless, these *in vivo* experiments accord with our *in vitro* results, which indicated that SurA has a larger effect on the rate of CdiA toxin cleavage. Furthermore, the fact that SurA influences the overall efficiency of CdiA-dependent killing in cells suggests that the toxin cleavage step may also be the slow step in the CDI process *in vivo*.

## Discussion

CDI systems have been shown to play roles in interbacterial competition and signaling, biofilm formation, and pathogenesis, and yet many of the molecular details of how these systems deliver their toxins into the cells they target remain unclear (14, 25, 77, 78). Here we have developed an assay system that allows us to analyze steps in that toxin delivery mechanism. To our knowledge, this is first *in vitro* reconstitution of any of the membrane-associated steps in the CDI process, and it allows us to answer questions that cannot be easily addressed *in vivo*.

Our results indicate that CdiA rapidly assembles into membranes in a manner that depends on BamA, but BamA does not appear to play an active role in that process. CdiA forms specific interactions with BamA’s extracellular surface but is insensitive to conformational changes in BamA’s lateral gate. The fact that the L4-L6 (S559C/Q678C) disulfide bond in BamA reduces the efficiency of CdiA cleavage nevertheless suggests that CdiA inserts into the OM near BamA’s lateral gate—in accord with our previous studies and with the AlphaFold2-predicted structure (31). The unusually short β-strands that form BamA’s lateral gate are known to distort the adjacent lipid bilayer, and this distortion could potentially facilitate CdiA’s insertion at these sites in the OM (36, 37, 40). In support of that model, our experiments indicate that the CdiA is highly sensitive to the lipid composition of the proteoliposomes; however, further investigation is needed to understand how CdiA assembles into a membrane that more closely resembles the OM, which includes lipopolysaccharides and different types of phospholipids. It should now be possible to evaluate how incorporating small amounts of these native lipids into the proteoliposomes alters the efficiency of CdiA membrane insertion and toxin cleavage. BamA’s role in facilitating CdiA toxin delivery by altering the physical properties of the OM might then be assessed.

The experiments presented here also suggest that periplasmic chaperones may facilitate toxin cleavage in target cells. Although CdiA inserts into the lipid bilayers in our experiments relatively quickly, toxin cleavage is comparatively slow. The VENN cleavage site residues, therefore, must not be properly arranged for autoproteolysis until late in the process. The slow assembly of the cleavage site may be advantageous in that it could prevent premature release of the toxin into the extracellular environment or into the target OM; however, the cleavage should occur when the toxin reaches its intended location in the target cell’s periplasm. Our experiments demonstrate that while SurA is covalently bound to BamA, it can directly interact with CdiA and increase the rate of toxin cleavage *in vitro*. SurA overexpression also renders target cells more sensitive to CDI. It is not yet clear how SurA facilitates toxin cleavage, but we propose that SurA interacts with CdiA as it emerges on the periplasmic side of the target OM. The chaperone might help the protein adopt the proper conformation for the VENN sequence to cleave the N2908-A2909 peptide bond. Alternatively, or concomitantly, SurA might assist in translocating the CT domain into the periplasm by binding to its hydrophobic regions. SurA has not previously been suggested to assist in a process that transports proteins from the OM into the periplasm, but Skp has been shown to remove OMPs from the Bam complex when they stall during their assembly (75). Perhaps the EC93 CDI system has evolved to make use of the native interactions between the Bam complex and these OMP chaperones to facilitate cleavage of its toxin.

Our experiments are consistent with several observations about how CDI systems function more broadly. The EC93 CDI system uses BamA as a receptor, but other CDI systems utilize other OMP receptors, which do not catalyze protein assembly. The absence of any direct role for BamA’s lateral gate in CdiA assembly is therefore not surprising; the FHA-2 domains of different CdiA proteins likely all assemble into the OMs of their targets by a similar mechanism. CDI systems also transport a variety of toxins that have different structures and that need to be transported into or across the target cell’s IM to exert their lethal effects. Periplasmic chaperones could potentially assist in those subsequent steps in the delivery process by keeping the toxins in an unfolded state as they are transported across the periplasm to their final destinations. Further characterization of the delivery mechanisms of other CDI systems is necessary to understand how different toxins are transported and whether they interact with chaperones or other factors in their targets. The methods we have developed here should enable such studies and should allow us to identify the conserved features of this unusual mechanism of protein transport in the gram-negative cell envelope.

## Experimental procedures

### General materials

The materials and equipment used in this study are described in the Supporting Information. Descriptions of the plasmids used for protein expression are provided in Table S1. In all purifications and procedures, tris-buffered saline (TBS) (pH 8.0) is defined as 20 mM Tris (pH 8.0) with 150 mM NaCl, and 2× SDS sample buffer is defined as 125 mM Tris (pH 6.8) with 4% (w/v) sodium dodecyl sulfate, 20% (v/v) glycerol, 0.04% (w/v) bromophenol blue, and 5% (v/v) β-mercaptoethanol. All buffers containing urea were prepared on the day of use.

### AlphaFold2 structure predictions

An AlphaFold2 structure of amino acids R1358-K3132 of *E. coli* EC93 CdiA bound to the BamA β-barrel (amino acids N422-W810) was generated using ColabFold (https://colab.research.google.com/github/sokrypton/ColabFold/blob/main/AlphaFold2.ipynb) (79, 80). The run parameters were as follows: template mode – none, msa mode – mmseqs2_uniref_env, pair mode – unpaired_paired, and model type – alphafold2_multimer_v3. Five models were generated and ranked by pLDDT score. The highest ranked model is shown in Figure 1*C*. All protein structure images were prepared with PyMOL 3.1.6.1.

### Flag-CdiA-CT-His expression and purification

The wildtype Flag-CdiA-CT-His fragment was expressed from plasmid pCH1237, which encodes amino acids R1358-K3132 ΔP1567-N1820 of the *E. coli* EC93 CdiA sequence with an N-terminal Flag-tag and C-terminal His_6_-tag. The W1519A and N2908A Flag-CdiA-CT-His fragments were expressed from plasmids pCH1240 and pCH1241, respectively. Starter cultures of *E. coli* BL21(DE3) cells carrying these plasmids were grown overnight at 30 °C with shaking at 220 rpm in LB media with 50 μg/ml kanamycin. The next day, 500 ml of fresh LB media containing 50 μg/ml kanamycin were inoculated with 5 ml of starter culture and then incubated at 37 °C and 220 rpm. When each culture reached an *A*_600_ = 0.5, protein expression was induced by adding isopropyl β-D-1-thiogalactopyranoside (IPTG) to a final concentration of 0.2 mM. The temperature was then lowered to 16 °C, and the cultures were incubated overnight with shaking at 220 rpm. The cells were then collected by centrifugation at 5250 × *g* for 15 min at 4 °C.

Each cell pellet was resuspended in 10 ml of TBS (pH 8.0) and then centrifuged again at 5250 × *g* for 15 min at 4 °C. Each pellet was then resuspended in 10 ml of BugBuster Protein Extraction Reagent supplemented with a complete ULTRA protease inhibitor cocktail without EDTA. Deoxyribonuclease I and lysozyme were each added to final concentrations of 0.2 mg/ml, and the resulting cell suspensions were incubated on a rocker at room temperature for approximately 1 h to lyse completely. The lysates were centrifuged at 5250 × *g* for 10 min at 4 °C, and the supernatants were removed by vacuum aspiration. The inclusion body pellets were washed by resuspension in 10 ml of TBS (pH 8.0) and then centrifuged again at 5250 × *g* for 10 min at 4 °C. The final pellets were then resuspended in 2 to 3 ml of 8 M urea. The resulting solutions were incubated on a rocker at room temperature for 30 to 60 min until the inclusion bodies dissolved and then centrifuged at 5250 × *g* for 10 min at 4 °C.

The urea-denatured protein in each supernatant was diluted ∼10-fold into 20 ml of TBS (pH 8.0) containing 1 M urea and then subjected to Ni-NTA affinity chromatography. Typically, 2 ml of packed Ni-NTA Superflow resin were pre-equilibrated with TBS (pH 8.0) containing 2 M urea and 20 mM imidazole. The resin was then incubated with the diluted protein solution for 2 h at room temperature with gentle end-over-end rotation. After collecting the flow through, the resin was washed twice with 20 ml of TBS (pH 8.0) containing 2 M urea and 20 mM imidazole. The protein was then eluted with 10 ml of TBS (pH 8.0) containing 2 M urea and 500 mM imidazole. Each eluate was concentrated in an Amicon Ultra 30 kDa molecular weight cutoff filter and then subjected to size-exclusion chromatography at 4 °C on a Superose 6 Increase 10/300 GL column in TBS (pH 8.0) containing 2 M urea. The Flag-CdiA-CT-His proteins eluted at approximately 11.8 ml; fractions from this peak were collected and concentrated in an Amicon Ultra 30 kDa molecular weight cutoff filter to a final concentration of approximately 5 mg/ml. The purified protein was flash frozen in liquid nitrogen and stored at −80 °C until use.

### BamA expression and purification

Wildtype *E. coli* BamA was expressed with an N-terminal His_6_-tag and without its signal sequence in BL21(DE3) cells carrying plasmid pCH740. The Y550N/E554Q/H555Y and disulfide-bonded variants of this His_6_-BamA construct were similarly produced from plasmids pCH1230, pCH746, pCH747, pCH748, pCH1189, pCH1197, and pCH1212. Cultures of these strains were grown in LB with 50 μg/ml carbenicillin at 37 °C with shaking at 220 rpm to an *A*_600_ = 0.4. Protein expression was then induced by adding IPTG to a concentration of 0.2 mM, and the cultures were incubated at 37 °C for another 4 h. The cells were collected by centrifugation at 5250 × *g* for 10 min at 4 °C. The pellets were resuspended in BugBuster Protein Extraction Reagent supplemented with a complete ULTRA protease inhibitor cocktail without EDTA. Deoxyribonuclease I and lysozyme were each added to final concentrations of 0.2 mg/ml, and the resulting cell suspensions were incubated on a rocker at room temperature for 30 to 60 min to lyse completely. The lysates were centrifuged at 5250 × *g* for 10 min at 4 °C, and the supernatants were removed by vacuum aspiration. The inclusion body pellets were then washed twice by resuspension in 10 ml of TBS (pH 8.0) and centrifugation at 5250 × *g* for 10 min at 4 °C. The final pellets were resuspended in 8 M urea and incubated on a rocker at room temperature for 30 to 60 min until the inclusion bodies dissolved. The resulting solutions were then centrifuged at 20,000 × *g* for 10 min at 4 °C to remove debris, and the supernatants were collected. Each urea-denatured protein was then diluted 10-fold into TBS (pH 8.0) containing 0.5% (w/v) n-dodecyl-N,N-dimethylamine-N-oxide and incubated on a rocker at room temperature overnight.

The next day, tris(1,10-phenanthroline)copper(II) sulfate (*i.e.*, [Cu(phen)_3_]SO_4_) was added to each refolded protein containing non-native cysteines to generate the disulfide-bonded variants of His_6_-BamA. A solution of 10 mM [Cu(phen)_3_]SO_4_ was prepared by dissolving 10 mM CuSO_4_ in water and adding 30 mM 1,10-phenanthroline. This stock solution was diluted into the refolded His_6_-BamA solutions to a final concentration of 200 μM [Cu(phen)_3_]SO_4_. These solutions were incubated on a rocker at room temperature for an additional 2 h to allow the proteins to oxidize. All of the His_6_-BamA proteins were then subjected to Ni-NTA affinity and size-exclusion chromatography. Each Ni-NTA gravity column was washed with TBS (pH 8.0) containing 0.05% (w/v) n-dodecyl-β-D-maltoside (DDM) and 20 mM imidazole, and the His_6_-BamA proteins were eluted with TBS (pH 8.0) containing 0.05% (w/v) DDM and 200 mM imidazole. The eluates were concentrated in Amicon Ultra 100 kDa molecular weight cutoff filters and then centrifuged at 20,000 × *g* for 10 min at 4 °C to remove any precipitated protein. The supernatants were subjected to size-exclusion chromatography on a Superdex 200 Increase 10/300 GL column in TBS (pH 8.0) containing 0.03% (w/v) DDM. Fractions containing the His_6_-BamA proteins were collected and concentrated in an Amicon Ultra 100 kDa molecular weight cutoff filters. The purified proteins were flash frozen in liquid nitrogen and stored at −80 °C until use.

Untagged versions of the wildtype and Y550N/E554Q/H555Y BamA proteins were produced in BL21(DE3) cells carrying plasmids pCH103 and pCH1183 by the same methods as described above. The proteins were also purified as described above except that the Ni-NTA affinity chromatography steps were omitted. After refolding the proteins in TBS (pH 8.0) containing 0.5% (w/v) n-dodecyl-N,N-dimethylamine-N-oxide overnight, and the solutions were concentrated and directly purified by size-exclusion chromatography.

### BamABCDE complex expression and purification

BL21(DE3) cells carrying plasmid pCH871, which encodes the five Bam proteins with a His_8_-tag on the C-terminal end of BamE, were grown overnight at 30 °C and 220 rpm in LB with 0.2% (w/v) glucose and 50 μg/ml carbenicillin. The starter culture was then diluted 100-fold into fresh LB with 50 μg/ml carbenicillin and incubated at 37 °C and 220 rpm. When the culture reached an *A*_600_ = 0.4, the temperature was lowered to 30 °C, and approximately 20 min later, IPTG was added to a concentration of 0.2 mM. The cultures were incubated for another 3.5 h at 30 °C, and the cells were then collected by centrifugation at 5250 × *g* for 15 min at 4 °C.

The cell pellets were resuspended in TBS (pH 8.0), and deoxyribonuclease I, lysozyme, and phenylmethylsulfonyl fluoride (PMSF) were added to concentrations of 0.2 mg/ml, 0.2 mg/ml, and 1 mM, respectively. The cells were lysed by three passes through a French Press. The resulting lysate was first centrifuged at 5250 × *g* for 10 min at 4 °C, and the supernatant was then ultracentrifuged at 40,000 rpm in a Type 45 Ti rotor (*i.e.*, ∼125,000 × *g*) for 45 min at 4 °C. The supernatant was discarded, and the membrane pellet was resuspended in TBS (pH 8.0) with 2% (v/v) Triton X-100 and 1 mM PMSF. This solution was incubated on a rocker at room temperature for approximately 1 h and then ultracentrifuged at 37,000 rpm in a Type 45 Ti rotor (*i.e.*, ∼107,000 × *g*) for 35 min at 4 °C. The supernatant was loaded on a Ni-NTA gravity column, which was pre-equilibrated in TBS (pH 8.0) containing 0.05% (w/v) DDM and 20 mM imidazole. After loading the protein solution, the column was washed with 20 column volumes of TBS (pH 8.0) containing 0.05% (w/v) DDM and 20 mM imidazole, and the Bam complex was eluted with four column volumes of TBS (pH 8.0) containing 0.05% (w/v) DDM and 200 mM imidazole. The eluate was concentrated in an Amicon Ultra 100 kDa molecular weight cutoff filter and then centrifuged at 20,000 × *g* for 10 min at 4 °C to remove any precipitated protein. The supernatant was subjected to size-exclusion chromatography on a Superdex 200 Increase 10/300 GL column in TBS (pH 8.0) containing 0.03% (w/v) DDM and 1 mM tris(2-carboxyethyl)phosphine hydrochloride. The Bam complex eluted at 11.6 ml; fractions collected from this peak were concentrated in an Amicon Ultra 100 kDa molecular weight cutoff filter, flash frozen in liquid nitrogen, and stored at −80 °C until use.

### Wildtype and K27C SurA expression and purification

The wildtype and K27C forms of SurA were produced in BL21(DE3) cells carrying plasmids pCH917 and pCH1272, respectively. These plasmids encode SurA’s mature sequence (amino acids 21–428) with an N-terminal His_6_-tag followed by a thrombin cleavage site. Starter cultures of these strains were grown overnight at 37 °C in LB with 50 μg/ml kanamycin. The next day, they were diluted 100-fold into fresh LB with 50 μg/ml kanamycin and incubated at 37 °C with shaking at 220 rpm. When the cultures reached an *A*_600_ = 0.4, IPTG was added to a concentration of 0.2 mM. After another 4 h of incubation at 37 °C, the cells were collected by centrifugation at 5250 × *g* for 15 min at 4 °C. The pellets were resuspended in TBS (pH 8.0), and deoxyribonuclease I, lysozyme, and PMSF were added to concentrations of 0.2 mg/ml, 0.2 mg/ml, and 1 mM, respectively. The cells were lysed by three passes through a French Press, and the lysates were centrifuged at 5250 × *g* for 10 min at 4 °C. The supernatants were then subjected to Ni-NTA chromatography. After loading the supernatants on Ni-NTA resin, each column was washed with 20 column volumes of TBS (pH 8.0) containing 20 mM imidazole, and the His_6_-SurA proteins were eluted with four column volumes of TBS (pH 8.0) containing 200 mM imidazole. The eluates were concentrated in an Amicon Ultra 30 kDa molecular weight cutoff filters.

Each concentrated protein was digested with restriction grade thrombin overnight at 4 °C. The next day, each protein was diluted 10-fold with TBS (pH 8.0) and subjected to Ni-NTA chromatography to remove the cleaved His_6_-tag and any undigested protein. The Ni-NTA resin was equilibrated with TBS (pH 8.0) containing 20 mM imidazole. The protein solution was loaded and the flow-through was collected and reloaded on the resin three times. The resin was then washed with five column volumes of TBS (pH 8.0) containing 20 mM imidazole. The final flow-through and wash fractions were combined and concentrated in an Amicon Ultra 30 kDa molecular weight cutoff filter. The protein was then subjected to size-exclusion chromatography on a Superdex 200 Increase 10/300 GL column in TBS (pH 8.0). Fractions from the peak at 14.3 ml were collected and concentrated in an Amicon Ultra 30 kDa molecular weight cutoff filter. The purified protein was flash frozen in liquid nitrogen and stored at −80 °C until use.

### SurA K27C/Q58pBPA expression and purification

SurA K27C/Q58*p*BPA was produced in BL21(DE3) cells carrying two plasmids: pEVOL-pBpF and pCH1284 (74). A starter culture was grown overnight at 37 °C in LB with 30 μg/ml chloramphenicol, 50 μg/ml kanamycin, and 0.2% (w/v) glucose. The next day, a stock solution of 0.9 M *p*BPA was prepared in 1 M NaOH and then diluted into prewarmed LB to a concentration of 0.9 mM. 30 μg/ml chloramphenicol and 50 μg/ml kanamycin were added, and the starter culture was diluted 100-fold into this media. The culture was incubated at 37 °C with shaking at 220 rpm until it reached an *A*_600_ = 0.4. Protein expression was then induced by adding 0.2 mM IPTG and 0.02% (w/v) arabinose, and the culture was incubated at 37 °C for another 3.5 h. The cells were collected by centrifugation at 5250 × *g* for 15 min at 4 °C. The cells were then lysed, and the SurA K27C/Q58*p*BPA protein was purified as described for the wildtype SurA protein except that the final size-exclusion chromatography step was omitted.

### BamABCDE–SurA complex purification

The BamABCDE complex containing an R76C substitution in BamA was expressed in BL21(DE3) cells carrying plasmid pCH1275 and purified by the same procedure as described for the wildtype BamABCDE complex above. The purified BamA(R76C)BCDE-His_8_ complex was then mixed with either purified SurA K27C or purified SurA K27C/Q58*p*BPA in a ratio of 50 μM Bam complex: 75 μM SurA. The cysteines were then oxidized by adding 40 μM [Cu(phen)_3_]SO_4._ and incubating the protein mixtures on rocker at room temperature for 1 h. The solutions were then diluted ∼20-fold with TBS (pH 8.0) containing 0.03% (w/v) DDM and reconcentrated in Amicon Ultra 100 kDa molecular weight cutoff filters to remove some of the [Cu(phen)_3_]SO_4_. The concentrated proteins were centrifuged at 20,000 × *g* for 10 min at 4 °C, and the supernatants were subjected to size-exclusion chromatography on a Superdex 200 Increase 10/300 GL column in TBS (pH 8.0) containing 0.03% (w/v) DDM. The BamA(R76C)BCDE-SurA(K27C) complex eluted at 11.3 ml, and the BamA(R76C)BCDE-SurA(K27C/Q58*p*BPA) complex eluted at 10.5 ml. Fractions from these peaks were concentrated in Amicon Ultra 100 kDa molecular weight cutoff filters, and the purified protein complexes were flash frozen in liquid nitrogen and stored at −80 °C until use.

### Skp expression and purification

BL21(DE3) cells carrying plasmid pCH01, which encodes Skp’s mature sequence (amino acids 21–161) with an N-terminal His_6_-tag followed by a thrombin cleavage site, were grown overnight in LB with 50 μg/ml kanamycin and 0.2% (w/v) glucose. The starter culture was then diluted 100-fold into LB with 50 μg/ml kanamycin and incubated at 37 °C with shaking at 220 rpm. Protein expression was induced with 0.2 mM IPTG when the culture reached an *A*_600_ = 0.8 to 1.0, and the culture was incubated overnight at 16 °C. The cells were collected by centrifugation at 5250 × *g* for 15 min at 4 °C. The pellets were resuspended in TBS (pH 8.0), and deoxyribonuclease I, lysozyme, and PMSF were added to concentrations of 0.2 mg/ml, 0.2 mg/ml, and 1 mM, respectively. The cells were lysed by three passes through a French Press, and the resulting lysate was centrifuged at 5250 × *g* for 15 min at 4 °C. The supernatant was then loaded on Ni-NTA resin that had been pre-equilibrated in TBS (pH 8.0) containing 40 mM imidazole. The resin was washed with 10 to 20 column volumes of TBS (pH 8.0) containing 40 mM imidazole, and His_6_-Skp was eluted with three column volumes of TBS (pH 8.0) with 200 mM imidazole. The eluate was concentrated in an Amicon Ultra 10 kDa molecular weight cutoff filter, and the buffer was exchanged by diluting the protein (∼10-fold) with TBS (pH 8.0) and re-concentrating. The purified protein was flash frozen in liquid nitrogen and stored at −80 °C until use.

### Proteoliposome preparation

Proteoliposomes containing the Bam proteins were prepared based on previously published methods (39, 81). Typically, 10 μM solutions of the wildtype or mutant forms of BamA, the BamABCDE complex, the BamA(R76C)BCDE-SurA(K27C) complex, or the BamA(R76C)BCDE-SurA(K27C/Q58pBPA) complex were prepared in TBS (pH 8.0) containing 0.03% (w/v) DDM. A 20 mg/ml suspension of DMPC phospholipids in water was also prepared and sonicated in a water bath at room temperature until it appeared translucent (*i.e.*, for ∼5 min). Forty microliters of the DMPC phospholipids were then added to 200 μl of the 10 μM protein solutions. A set of empty liposomes were simultaneously prepared by adding 40 μl of the DMPC phospholipids to 200 μl of TBS (pH 8.0) containing 0.03% (w/v) DDM. These mixtures were incubated on ice for 5 min and then diluted with 8 ml of TBS (pH 8.0). After 30 min of incubation on ice, the solutions were ultracentrifuged in a Type 90 Ti rotor at 63,000 rpm (*i.e.*, ∼250,00*×g*) for 2 h. The supernatants were vacuum aspirated, and the pellets were resuspended in 200 μl of TBS (pH 8.0). These proteoliposome solutions were flash frozen in liquid nitrogen and stored at −80 °C until use.

For the sedimentation assay in Figure 4, proteoliposomes containing a higher concentration of BamA were prepared by adding 40 μl of sonicated DMPC lipids to 200 μl of a 25 μM solution of either the wildtype or Y550N/E554Q/H555Y His_6_-BamA. These mixtures were then diluted and ultracentrifuged as described above, and the pellets were resuspended in 200 μl of TBS (pH 8.0).

To prepare proteoliposomes containing SurA and Skp for the experiments in Figure 5, DMPC lipids were added to 10 μM solutions of the BamABCDE or BamA(R76C)BCDE-SurA(K27C) complexes as described above. These mixtures were then diluted with 8 ml of a 10 μM solution of purified SurA or 8 ml of a 30 μM solution of purified His_6_-Skp in TBS (pH 8.0). The 30 μM His_6_-Skp concentration was calculated based on the molecular weight of a His_6_-Skp monomer. After 30 min on ice, the solutions were ultracentrifuged as described above, and the pelleted proteoliposomes were resuspended in 200 μl of TBS (pH 8.0).

Proteoliposomes composed of other phospholipids were prepared for the experiment in Figure S3 by these same methods. The lipids listed in Figure S3 were all prepared as 20 mg/ml suspensions in water and added to 10 μM solutions of wildtype BamA.

### Flag-CdiA-CT-His cleavage assays in proteoliposomes

Empty liposomes and proteoliposomes containing ∼10 μM Bam proteins were sonicated in a water bath at room temperature in 10 s intervals until the solutions appeared homogenous and translucent. To prepare a typical 20 μl reaction, 5 μl of the proteoliposomes were then added to 11 μl of TBS (pH 8.0). The purified Flag-CdiA-CT-His proteins were diluted from their stock solutions into TBS (pH 8.0) to a concentration of 5 μM. Four microliters of these 5 μM solutions were then added to the proteoliposome solutions such that the final concentrations of the Flag-CdiA-CT-His and Bam proteins were 1 μM and ∼2.5 μM, respectively, in the reaction mixtures. These mixtures were incubated at 25 °C for the time periods indicated in the figures and then quenched by adding an equal volume of 2x SDS sample buffer and boiling for 10 min at 95 °C. For the time course experiments, larger reaction volumes were prepared with the same concentrations of Flag-CdiA-CT-His and proteoliposomes. Ten microliter aliquots were removed at the time points indicated in the figures, quenched, and stored a −20 °C until all of the samples were collected.

The quenched samples were subjected to SDS-PAGE on 4 to 20% TGX gels at 200 V for 40 min. The gels were stained with Coomassie Blue or transferred to low fluorescence PVDF membranes on a Bio-Rad Trans-Blot Turbo Transfer System at a constant current of 2.5 A for 3 min. The membranes were incubated overnight at 4 °C with anti-Flag DyLight 680 and anti-His CF 488A antibodies at dilutions of 1:5000. The membranes were then imaged using the DyLight 680 and DyLight 488 filters on a Bio-Rad ChemiDoc MP system. Cleavage efficiencies were determined by quantifying the densities of the Flag-CdiA-CT-His (uncleaved) and Flag-CdiA (cleaved) bands in the DyLight 680 images using the Bio-Rad Image Lab 6.0 software.

### Flag-CdiA-CT-His sedimentation assay in proteoliposomes

Proteoliposomes containing ∼25 μM wildtype His_6_-BamA or Y550N/E554Q/H555Y His_6_-BamA were sonicated in a water bath at room temperature in 10 s intervals until the solutions appeared homogenous and translucent. One-hundred twenty microliter reactions were then prepared by diluting 30 μl of the proteoliposome stock solutions into 66 μl of TBS (pH 8.0). Purified Flag-CdiA-CT-His was diluted to 5 μM in TBS (pH 8.0), and then 24 μl of the 5 μM solution were added to each reaction. The final concentrations of the Flag-CdiA-CT-His and His_6_-BamA proteins in the reaction mixtures were 1 μM and ∼6.25 μM, respectively. Ten microliter aliquots were removed from the reactions immediately after the Flag-CdiA-CT-His was added (t = 0) and at the endpoints indicated in the figures (t = 0.5 or 4 h). These aliquots were quenched by adding an equal volume of 2x SDS sample buffer and boiling for 10 min at 95 °C. The remaining 100 μl of each reaction were centrifuged at 25,000 × *g* for 60 min at 4 °C. Samples of the supernatants were removed and quenched, and the pellets were resuspended in 100 μl of ice-cold 100 mM Na_2_CO_3_. A 10 μl sample of each resuspended pellet was removed and quenched, and the remainder was incubated on ice for 30 min and then centrifuged at 25,000 × *g* for 60 min at 4 °C. Samples of the supernatants were again collected and quenched, and the pellets were resuspended in 90 μl of TBS (pH 8.0) and quenched.

The quenched samples were subjected to SDS-PAGE on 4 to 20% TGX gels at 200 V for 40 min and transferred to low fluorescence PVDF membranes on a Bio-Rad Trans-Blot Turbo Transfer System at a constant current of 2.5 A for 3 min. The membranes were incubated overnight at 4 °C with anti-Flag DyLight 680 and anti-His CF 488A antibodies at dilutions of 1:5000. The membranes were then imaged using the DyLight 680 and DyLight 488 filters on a Bio-Rad Chemi-Doc MP system. The Bio-Rad Image Lab 6.0 software was used to determine the densities of the His_6_-BamA, Flag-CdiA-CT-His (uncleaved), and Flag-CdiA (cleaved) bands. The densities of the Flag-CdiA-CT-His and Flag-CdiA bands in each sample were summed and then divided by the density of the His_6_-BamA band in the same sample to determine the ratio of CdiA: BamA present. The CdiA: BamA ratio in the final postwash pellet sample was then divided by the ratio in the precentrifugation reaction endpoint sample (t = 0.5 or t = 4) to determine what percentage of the CdiA in the reaction mixture cosedimented with BamA in the final proteoliposome pellet.

### SurA photocrosslinking in proteoliposomes

Proteoliposomes containing ∼10 μM BamA(R76C)BCDE-SurA(K27C/Q58*p*BPA) complex were sonicated in a water bath at room temperature in 10 s intervals until the solution appeared homogenous and translucent. Fifty microliter reactions were then prepared by diluting 12.5 μl of the proteoliposome stock solution into 27.5 μl of TBS (pH 8.0). The purified wildtype and mutant Flag-CdiA-CT-His proteins were diluted to 5 μM in TBS (pH 8.0), and then 10 μl of these diluted solutions were added to the proteoliposomes. The final concentrations of the Flag-CdiA-CT-His and Bam–SurA complexes in the reaction mixtures were 1 μM and ∼2.5 μM, respectively. The reaction mixtures were incubated at 25 °C for 60 min, 25 μl were then removed from each reaction, placed on ice, and protected from light. The remaining 25 μl of each reaction were placed on ice and irradiated with 365 nm light for 20 min using a 100 W UVP High-Intensity UV Inspection Lamp (Model B-100AP). The unirradiated (-UV) and irradiated (+UV) samples were then quenched with 25 μl of 2x SDS sample buffer and boiled at 95 °C for 10 min.

The quenched samples were subjected to SDS-PAGE on 4 to 20% TGX gels at 200 V for 40 min. The gels were then stained with Coomassie Blue or transferred to low fluorescence PVDF membranes on a Bio-Rad Trans-Blot Turbo Transfer System at a constant current of 2.5 A for 3 min. The membranes were incubated overnight at 4 °C with anti-Flag DyLight 680 and anti-His CF 488A antibodies at dilutions of 1:5000. The membranes were then imaged using the DyLight 680 and DyLight 488 filters on a Bio-Rad Chemi-Doc MP system. The densities of the noncrosslinked Flag-CdiA-CT-His (uncleaved) and Flag-CdiA (cleaved) bands in the DyLight 680 images were quantified using Bio-Rad Image Lab 6.0 software. The densities of these bands in each sample were summed, and the total density in each +UV sample was compared to the total in the corresponding -UV sample to determine the percentage of CdiA that remains noncrosslinked upon irradiation.

### Competition assays with chaperone-expressing target cells

The full-length wildtype and N2908A forms of CdiA were produced in cells carrying plasmids pCH566 and pCH1145, respectively. These plasmids contain the *E. coli* EC93 *cdiBAI* genes with an insertion in *cdiA* encoding a 3xFLAG tag between amino acids Q643 and G644 and the appropriate mutation to generate the N2908A substitution. KRX cells carrying each of these plasmids were grown overnight at 30 °C with shaking at 220 rpm in LB containing 50 μg/ml carbenicillin and 0.2% glucose. The SurA, Skp, and MBP proteins were produced from plasmids pCH1295, pCH1309, and pCH131, respectively. KRX carrying each of these plasmids or pET-26b (the empty vector) were grown overnight at 30 °C with shaking at 220 rpm in LB containing 50 μg/ml kanamycin and 0.2% glucose. The next day, 50 μl of each of the starter cultures were transferred into 5 ml of terrific broth without antibiotics. These cultures were grown at 37 °C with shaking at 220 rpm to an *A*_600_ = 0.6 to 0.7. The inhibitor cell cultures were then mixed with each of the target cell cultures in a 1:1 ratio and L-rhamnose, and IPTG were added to concentrations of 0.1% (w/v) and 0.2 mM, respectively. The mixed cell cultures were then incubated at 30 °C with shaking at 220 rpm for 4 h. Samples of each culture were removed immediately after mixing the inhibitor and target cells and after the 4-h incubation period. These samples were serially diluted by 10-fold in LB to a final dilution of 10^-5^. 2.5 μl samples of each dilution were plated on LB agar plates containing 50 μg/ml carbenicillin or 50 μg/ml kanamycin. The plates were incubated at 37 °C overnight.

### Trypsin digestion of Bam proteoliposomes

BamA’s orientation in the proteoliposome membranes was analyzed by a protease protection assay that was adapted from previously published procedures (44, 82). Proteoliposomes containing ∼10 μM His_6_-BamA, BamABCDE, or BamA(R76C)BCDE-SurA(K27C) were diluted into TBS (pH 8.0) or TBS (pH 8.0) containing 1% (w/v) DDM. Trypsin was then added to a final concentration of 25 μg/ml. The final concentrations of the Bam proteins in the reaction mixtures were ∼2.5 μM. The reactions were incubated at 37 °C, and aliquots were removed every 30 min. The aliquots were immediately quenched by mixing with an equal volume of 2x SDS sample buffer containing 2 mM PMSF and boiling at 95 °C for 10 min. The quenched samples were subjected to SDS-PAGE on 4 to 20% TGX gels at 200 V for 40 min and then Coomassie stained or transferred to low fluorescence PVDF membranes on a Bio-Rad Trans-Blot Turbo Transfer System at a constant current of 2.5 A for 3 min. The membranes were incubated with rabbit anti-BamA antibodies, which recognize BamA’s POTRA domains (20), at a dilution of 1:50,000 for 1 h at room temperature. The membranes were then washed three times with TBS (pH 8.0) containing 0.1% (v/v) Tween-20 for 10 min each and subsequently incubated with anti-rabbit DyLight 488 antibodies at a dilution of 1:10,000 for 1 h at room temperature. The membranes were then washed again and imaged using the DyLight 488 filter on a Bio-Rad Chemi-Doc MP system. The Bio-Rad Image Lab 6.0 software was used to determine the densities of the full-length BamA bands, and the percentage of BamA remaining at each time point was calculated by comparing the intensity of the BamA band in the digested (+trypsin) sample to that in the undigested (-trypsin) sample in the same immunoblot.

### CdiA RBD expression and purification

The wildtype CdiA RBD was produced from plasmid pCH933, which encodes amino acids R1358-G1573 of the *E. coli* EC93 CdiA sequence with a C-terminal His_6_-tag. Plasmid pCH1032 encodes this same construct with the W1519A substitution. BL21(DE3) cells carrying each of these plasmids were grown overnight in LB with 50 μg/ml kanamycin at 30 °C with shaking at 220 rpm. The next day, the starter cultures were diluted 100-fold into LB with 50 μg/ml kanamycin, and these cultures were incubated at 37 °C until they reached an *A*_600_ = 0.5. Protein expression was induced with 0.2 mM IPTG, and the cultures were incubated overnight at 16 °C. The cells were then collected by centrifugation at 5250 × *g* for 15 min at 4 °C. The pellets were resuspended in TBS (pH 8.0), and deoxyribonuclease I, lysozyme, and PMSF were added to concentrations of 0.2 mg/ml, 0.2 mg/ml, and 1 mM, respectively. The cells were lysed by three passes through a French Press, and the resulting lysates were centrifuged at 5250 × *g* for 15 min at 4 °C. The supernatants were then loaded on Ni-NTA resin that had been pre-equilibrated in TBS (pH 8.0) containing 20 mM imidazole. The resin was washed with 20 to 30 column volumes of TBS (pH 8.0) containing 20 mM imidazole, and the CdiA RBD-His_6_ proteins were eluted with nine column volumes of TBS (pH 8.0) with 200 mM imidazole. The eluates were concentrated in Amicon Ultra 10 kDa molecular weight cutoff filters and subjected to size-exclusion chromatography on a Superdex 200 Increase 10/300 GL column in TBS (pH 8.0). The CdiA RBD-His_6_ proteins eluted at 15.4 ml. Fractions from this peak were concentrated in Amicon Ultra 10 kDa molecular weight cutoff filters, flash frozen in liquid nitrogen, and stored at −80 °C until use.

An untagged version of the CdiA RBD was also prepared by similar methods. Plasmid pCH936 encodes amino acids R1358-G1573 of the CdiA sequence with an N-terminal His_6_-tag followed by an HRV-3C protease site. This construct was produced in BL21(DE3) cells and purified by Ni-NTA affinity chromatography as described above. The protein was concentrated in an Amicon Ultra 10 kDa molecular weight cutoff filter and then digested with HRV-3C protease overnight at 4 °C. The next day, the protein was diluted 10-fold with TBS (pH 8.0) and subjected to Ni-NTA chromatography to remove the cleaved His_6_-tag and any undigested protein. The Ni-NTA resin was equilibrated with TBS (pH 8.0) containing 20 mM imidazole. The protein solution was loaded, and the flow-through was collected and reloaded on the resin three times. The resin was then washed with four column volumes of TBS (pH 8.0) containing 20 mM imidazole. The final flow-through and wash fractions were combined and concentrated in an Amicon Ultra 10 kDa molecular weight cutoff filter. The protein was then subjected to size-exclusion chromatography as described above.

### BamA and CdiA RBD copurification assays

The His_6_-tagged wildtype and W1519A CdiA RBD proteins were mixed with untagged wildtype and Y550N/E554Q/H555Y BamA proteins in a 1:3 M ratio—that is, solutions containing 5 μM CdiA RBD-His_6_ and 15 μM BamA were prepared in TBS (pH 8.0) containing 0.03% (w/v) DDM in a total volume of 220 μl. Control samples containing the BamA proteins without any CdiA RBD-His_6_ proteins were also prepared. These protein solutions were incubated on ice for 10 min, and a 20 μl “input” sample was removed from each and mixed with 20 μl of 2x SDS sample buffer. The remaining 200 μl of each protein solution were subjected to Ni-NTA affinity chromatography. The proteins were loaded on Ni-NTA resin that had been pre-equilibrated in TBS (pH 8.0) containing 0.05% (w/v) DDM and 20 mM imidazole. After loading the samples on the resin, it was washed three times with 10 column volumes of TBS (pH 8.0) containing 0.05% (w/v) DDM and 20 mM imidazole. The bound proteins were then eluted with three column volumes of TBS (pH 8.0) containing 0.05% (w/v) DDM and 200 mM imidazole. Samples of each eluate were removed and mixed with 2× SDS sample buffer. The “input” and “eluate” samples were boiled at 95 °C for 10 min and then subjected to SDS-PAGE on a 4 to 20% TGX gel at 200 V for 40 min. The gel was stained with Coomassie Blue.

The His_6_-tagged wildtype and S559C/Q678C BamA proteins were mixed with untagged CdiA RBD in a 1:3 M ratio (*i.e.*, 5 μM His_6_-BamA with 15 μM CdiA RBD). Control samples without any His_6_-BamA proteins were similarly prepared. These solutions were subjected to Ni-NTA affinity chromatography as described above. Two sets of “input” and “eluate” samples were prepared; one set with the standard 2x SDS sample buffer containing β-mercaptoethanol and one set with 2x SDS sample buffer lacking β-mercaptoethanol. All of the samples were boiled and subjected to SDS-PAGE and Coomassie staining as described above.

### Analysis of CdiA cleavage rates

The Flag-CdiA-CT-His cleavage rates were approximated by fitting the data in Figure 5*C* with one phase association kinetics using Prism 11. The data were modeled with an equation of the form $y=y_{0}+y_{\infty }\left(1-e^{-\text{kx}}\right)$ where $y_{0}$ was constrained to zero, and $y_{\infty }$ was constrained to 100, and k is the rate constant. The half-lives in Figure S6*B* were calculated as ln (2)/k.

## Data availability

All data are provided within this manuscript.

## Supporting information

This article contains supporting information (11, 26, 31, 51, 72, 73, 74, 82, 83, 84).

## Conflict of interest

The authors declare that they have no conflicts of interest with the contents of this article.

## References

[1] Garcia-Bayona L., Comstock L.E. (2018). Bacterial antagonism in host-associated microbial communities. Science.

[2] Ruhe Z.C., Low D.A., Hayes C.S. (2020). Polymorphic toxins and their immunity proteins: diversity, evolution, and mechanisms of delivery. Annu. Rev. Microbiol..

[3] Peterson S.B., Bertolli S.K., Mougous J.D. (2020). The central role of interbacterial antagonism in bacterial life. Curr. Biol..

[4] Cascales E., Buchanan S.K., Duche D., Kleanthous C., Lloubes R., Postle K. (2007). Colicin biology. Microbiol. Mol. Biol. Rev..

[5] Housden N.G., Hopper J.T., Lukoyanova N., Rodriguez-Larrea D., Wojdyla J.A., Klein A. (2013). Intrinsically disordered protein threads through the bacterial outer-membrane porin OmpF. Science.

[6] Lee S., Housden N.G., Ionescu S.A., Zimmer M.H., Kaminska R., Kleanthous C. (2020). Transmembrane epitope delivery by passive protein threading through the pores of the OmpF porin trimer. J. Am. Chem. Soc..

[7] Williams-Jones D.P., Webby M.N., Press C.E., Gradon J.M., Armstrong S.R., Szczepaniak J. (2023). Tunable force transduction through the Escherichia coli cell envelope. Proc. Natl. Acad. Sci. U. S. A..

[8] Cherrak Y., Flaugnatti N., Durand E., Journet L., Cascales E. (2019). Structure and activity of the type VI secretion System. Microbiol. Spectr..

[9] Wohlfarth J.C., Ward D., Pereira J., Basler M. (2026). The type VI secretion system and associated effector proteins. Nat. Rev. Microbiol..

[10] Aoki S.K., Pamma R., Hernday A.D., Bickham J.E., Braaten B.A., Low D.A. (2005). Contact-dependent inhibition of growth in Escherichia coli. Science.

[11] Aoki S.K., Diner E.J., de Roodenbeke C.T., Burgess B.R., Poole S.J., Braaten B.A. (2010). A widespread family of polymorphic contact-dependent toxin delivery systems in bacteria. Nature.

[12] Nikolakakis K., Amber S., Wilbur J.S., Diner E.J., Aoki S.K., Poole S.J. (2012). The toxin/immunity network of Burkholderia pseudomallei contact-dependent growth inhibition (CDI) systems. Mol. Microbiol..

[13] Willett J.L., Ruhe Z.C., Goulding C.W., Low D.A., Hayes C.S. (2015). Contact-Dependent Growth Inhibition (CDI) and CdiB/CdiA two-partner secretion proteins. J. Mol. Biol..

[14] Garcia E.C., Perault A.I., Marlatt S.A., Cotter P.A. (2016). Interbacterial signaling via Burkholderia contact-dependent growth inhibition system proteins. Proc. Natl. Acad. Sci. U. S. A..

[15] Cuthbert B.J., Hayes C.S., Goulding C.W. (2022). Functional and structural diversity of bacterial contact-dependent growth inhibition effectors. Front. Mol. Biosci..

[16] Mazar J., Cotter P.A. (2007). New insight into the molecular mechanisms of two-partner secretion. Trends Microbiol..

[17] Guerin J., Bigot S., Schneider R., Buchanan S.K., Jacob-Dubuisson F. (2017). Two-Partner secretion: combining efficiency and simplicity in the secretion of large proteins for bacteria-host and bacteria-bacteria interactions. Front. Cell. Infect. Microbiol..

[18] Guerin J., Botos I., Zhang Z., Lundquist K., Gumbart J.C., Buchanan S.K. (2020). Structural insight into toxin secretion by contact-dependent growth inhibition transporters. Elife.

[19] Ruhe Z.C., Subramanian P., Song K., Nguyen J.Y., Stevens T.A., Low D.A. (2018). Programmed secretion arrest and receptor-triggered toxin export during antibacterial contact-dependent growth inhibition. Cell.

[20] Aoki S.K., Malinverni J.C., Jacoby K., Thomas B., Pamma R., Trinh B.N. (2008). Contact-dependent growth inhibition requires the essential outer membrane protein BamA (YaeT) as the receptor and the inner membrane transport protein AcrB. Mol. Microbiol..

[21] Beck C.M., Willett J.L., Cunningham D.A., Kim J.J., Low D.A., Hayes C.S. (2016). CdiA effectors from uropathogenic Escherichia coli use heterotrimeric osmoporins as receptors to recognize target bacteria. Plos Pathog..

[22] Ruhe Z.C., Nguyen J.Y., Xiong J., Koskiniemi S., Beck C.M., Perkins B.R. (2017). CdiA effectors use modular receptor-binding domains to recognize target bacteria. mBio.

[23] Halvorsen T.M., Garza-Sanchez F., Ruhe Z.C., Bartelli N.L., Chan N.A., Nguyen J.Y. (2021). Lipidation of class IV CdiA Effector proteins promotes target cell recognition during contact-dependent growth inhibition. mBio.

[24] Ruhe Z.C., Wallace A.B., Low D.A., Hayes C.S. (2013). Receptor polymorphism restricts contact-dependent growth inhibition to members of the same species. mBio.

[25] Costello M.S., Neumann B., Raimondi M.W., Cuthbert B.J., Holubova J., Garza-Sanchez F. (2026). Bacteria deliver a microtubule-binding protein into mammalian cells to promote colonization. Science.

[26] Tiu A.K.Y., Conroy G.C., Bobst C.E., Hagan C.L. (2024). Autoproteolytic mechanism of CdiA toxin release reconstituted in vitro. J. Bacteriol..

[27] Halvorsen T.M., Schroeder K.A., Jones A.M., Hammarlof D., Low D.A., Koskiniemi S. (2024). Contact-dependent growth inhibition (CDI) systems deploy a large family of polymorphic ionophoric toxins for inter-bacterial competition. Plos Genet..

[28] Nakae T. (1975). Outer membrane of Salmonella typhimurium: reconstitution of sucrose-permeable membrane vesicles. Biochem. Biophys. Res. Commun..

[29] Nikaido H. (2003). Molecular basis of bacterial outer membrane permeability revisited. Microbiol. Mol. Biol. Rev..

[30] Benn G., Mikheyeva I.V., Inns P.G., Forster J.C., Ojkic N., Bortolini C. (2021). Phase separation in the outer membrane of Escherichia coli. Proc. Natl. Acad. Sci. U. S. A..

[31] Curley C.L., Fedrigoni T.P., Flaherty E.M., Woodilla C.J., Hagan C.L. (2021). Bacterial contact-dependent inhibition protein binds near the open lateral Gate in BamA prior to toxin translocation. Biochemistry.

[32] Voulhoux R., Bos M.P., Geurtsen J., Mols M., Tommassen J. (2003). Role of a highly conserved bacterial protein in outer membrane protein assembly. Science.

[33] Wu T., Malinverni J., Ruiz N., Kim S., Silhavy T.J., Kahne D. (2005). Identification of a multicomponent complex required for outer membrane biogenesis in Escherichia coli. Cell.

[34] Tomasek D., Kahne D. (2021). The assembly of beta-barrel outer membrane proteins. Curr. Opin. Microbiol..

[35] Birtles D., Fenn K.L., Machin J.M., Radford S.E., Ranson N.A. (2026). Integration of membrane proteins into the outer membrane of diderm bacteria by the BAM complex. Chem. Rev..

[36] Noinaj N., Kuszak A.J., Gumbart J.C., Lukacik P., Chang H., Easley N.C. (2013). Structural insight into the biogenesis of beta-barrel membrane proteins. Nature.

[37] Iadanza M.G., Schiffrin B., White P., Watson M.A., Horne J.E., Higgins A.J. (2020). Distortion of the bilayer and dynamics of the BAM complex in lipid nanodiscs. Commun. Biol..

[38] Liu J., Gumbart J.C. (2020). Membrane thinning and lateral gating are consistent features of BamA across multiple species. Plos Comput. Biol..

[39] Hagan C.L., Kim S., Kahne D. (2010). Reconstitution of outer membrane protein assembly from purified components. Science.

[40] Gessmann D., Chung Y.H., Danoff E.J., Plummer A.M., Sandlin C.W., Zaccai N.R. (2014). Outer membrane beta-barrel protein folding is physically controlled by periplasmic lipid head groups and BamA. Proc. Natl. Acad. Sci. U. S. A..

[41] Roman-Hernandez G., Peterson J.H., Bernstein H.D. (2014). Reconstitution of bacterial autotransporter assembly using purified components. Elife.

[42] Huysmans G.H., Guilvout I., Chami M., Nickerson N.N., Pugsley A.P. (2015). Lipids assist the membrane insertion of a BAM-independent outer membrane protein. Sci. Rep..

[43] Schiffrin B., Calabrese A.N., Higgins A.J., Humes J.R., Ashcroft A.E., Kalli A.C. (2017). Effects of periplasmic chaperones and membrane thickness on BamA-Catalyzed outer-membrane protein folding. J. Mol. Biol..

[44] Hussain S., Bernstein H.D. (2018). The Bam complex catalyzes efficient insertion of bacterial outer membrane proteins into membrane vesicles of variable lipid composition. J. Biol. Chem..

[45] Machin J.M., Kalli A.C., Ranson N.A., Radford S.E. (2023). Protein-lipid charge interactions control the folding of outer membrane proteins into asymmetric membranes. Nat. Chem..

[46] Nilaweera T.D., Brandes N.T., LaCroix I.S., Schwarz B., Bernstein H.D. (2026). Phospholipid composition strongly affects the assembly of beta barrel proteins into purified bacterial outer membranes. Nat. Commun..

[47] Pogozheva I.D., Tristram-Nagle S., Mosberg H.I., Lomize A.L. (2013). Structural adaptations of proteins to different biological membranes. Biochim. Biophys. Acta.

[48] Wu E.L., Fleming P.J., Yeom M.S., Widmalm G., Klauda J.B., Fleming K.G. (2014). E. coli outer membrane and interactions with OmpLA. Biophys. J..

[49] Storek K.M., Auerbach M.R., Shi H., Garcia N.K., Sun D., Nickerson N.N. (2018). Monoclonal antibody targeting the beta-barrel assembly machine of Escherichia coli is bactericidal. Proc. Natl. Acad. Sci. U. S. A..

[50] Iadanza M.G., Higgins A.J., Schiffrin B., Calabrese A.N., Brockwell D.J., Ashcroft A.E. (2016). Lateral opening in the intact beta-barrel assembly machinery captured by cryo-EM. Nat. Commun..

[51] Tomasek D., Rawson S., Lee J., Wzorek J.S., Harrison S.C., Li Z. (2020). Structure of a nascent membrane protein as it folds on the BAM complex. Nature.

[52] Wu R., Bakelar J.W., Lundquist K., Zhang Z., Kuo K.M., Ryoo D. (2021). Plasticity within the barrel domain of BamA mediates a hybrid-barrel mechanism by BAM. Nat. Commun..

[53] Doyle M.T., Jimah J.R., Dowdy T., Ohlemacher S.I., Larion M., Hinshaw J.E. (2022). Cryo-EM structures reveal multiple stages of bacterial outer membrane protein folding. Cell.

[54] Shen C., Chang S., Luo Q., Chan K.C., Zhang Z., Luo B. (2023). Structural basis of BAM-mediated outer membrane beta-barrel protein assembly. Nature.

[55] Thomson B.D., Marquez M.D., Rawson S., Dos Santos T.M.A., Harrison S.C., Kahne D. (2026). Structures of folding intermediates on BAM show diverse substrates fold by a conserved mechanism. Proc. Natl. Acad. Sci. U. S. A..

[56] Gu Y., Li H., Dong H., Zeng Y., Zhang Z., Paterson N.G. (2016). Structural basis of outer membrane protein insertion by the BAM complex. Nature.

[57] Noinaj N., Kuszak A.J., Balusek C., Gumbart J.C., Buchanan S.K. (2014). Lateral opening and exit pore formation are required for BamA function. Structure.

[58] White P., Haysom S.F., Iadanza M.G., Higgins A.J., Machin J.M., Whitehouse J.M. (2021). The role of membrane destabilisation and protein dynamics in BAM catalysed OMP folding. Nat. Commun..

[59] Wzorek J.S., Lee J., Tomasek D., Hagan C.L., Kahne D.E. (2017). Membrane integration of an essential beta-barrel protein prerequires burial of an extracellular loop. Proc. Natl. Acad. Sci. U. S. A..

[60] Molloy M.P. (2008). Isolation of bacterial cell membranes proteins using carbonate extraction. Methods Mol. Biol..

[61] Aoki S.K., Webb J.S., Braaten B.A., Low D.A. (2009). Contact-dependent growth inhibition causes reversible metabolic downregulation in Escherichia coli. J. Bacteriol..

[62] Combs A.N., Silhavy T.J. (2024). Periplasmic chaperones: outer membrane biogenesis and envelope stress. Annu. Rev. Microbiol..

[63] Devlin T., Fleming K.G. (2024). A team of chaperones play to win in the bacterial periplasm. Trends Biochem. Sci..

[64] Rouviere P.E., Gross C.A. (1996). SurA, a periplasmic protein with peptidyl-prolyl isomerase activity, participates in the assembly of outer membrane porins. Genes Dev..

[65] Rizzitello A.E., Harper J.R., Silhavy T.J. (2001). Genetic evidence for parallel pathways of chaperone activity in the periplasm of Escherichia coli. J. Bacteriol..

[66] Sklar J.G., Wu T., Kahne D., Silhavy T.J. (2007). Defining the roles of the periplasmic chaperones SurA, Skp, and DegP in Escherichia coli. Genes Dev..

[67] Vertommen D., Ruiz N., Leverrier P., Silhavy T.J., Collet J.F. (2009). Characterization of the role of the Escherichia coli periplasmic chaperone SurA using differential proteomics. Proteomics.

[68] Denoncin K., Schwalm J., Vertommen D., Silhavy T.J., Collet J.F. (2012). Dissecting the Escherichia coli periplasmic chaperone network using differential proteomics. Proteomics.

[69] Fenn K.L., Horne J.E., Crossley J.A., Bohringer N., Horne R.J., Schaberle T.F. (2024). Outer membrane protein assembly mediated by BAM-SurA complexes. Nat. Commun..

[70] Lehner P.A., Degen M., Jakob R.P., Modaresi S.M., Callon M., Burmann B.M. (2025). Architecture and conformational dynamics of the BAM-SurA holo insertase complex. Sci. Adv..

[71] Schiffrin B., Machin J.M., Karamanos T.K., Zhuravleva A., Brockwell D.J., Radford S.E. (2022). Dynamic interplay between the periplasmic chaperone SurA and the BAM complex in outer membrane protein folding. Commun. Biol..

[72] Chin J.W., Martin A.B., King D.S., Wang L., Schultz P.G. (2002). Addition of a photocrosslinking amino acid to the genetic code of Escherichia coli. Proc. Natl. Acad. Sci. U. S. A..

[73] Ryu Y., Schultz P.G. (2006). Efficient incorporation of unnatural amino acids into proteins in Escherichia coli. Nat. Methods.

[74] Young T.S., Ahmad I., Yin J.A., Schultz P.G. (2010). An enhanced system for unnatural amino acid mutagenesis in E. coli. J. Mol. Biol..

[75] Combs A.N., Silhavy T.J. (2022). The sacrificial adaptor protein Skp functions to remove stalled substrates from the beta-barrel assembly machine. Proc. Natl. Acad. Sci. U. S. A..

[76] Grabowicz M., Koren D., Silhavy T.J. (2016). The CpxQ sRNA negatively regulates skp to prevent mistargeting of beta-Barrel outer membrane proteins into the cytoplasmic membrane. mBio.

[77] Ruhe Z.C., Townsley L., Wallace A.B., King A., Van der Woude M.W., Low D.A. (2015). CdiA promotes receptor-independent intercellular adhesion. Mol. Microbiol..

[78] Allen J.P., Ozer E.A., Minasov G., Shuvalova L., Kiryukhina O., Satchell K.J.F. (2020). A comparative genomics approach identifies contact-dependent growth inhibition as a virulence determinant. Proc. Natl. Acad. Sci. U. S. A..

[79] Jumper J., Evans R., Pritzel A., Green T., Figurnov M., Ronneberger O. (2021). Highly accurate protein structure prediction with AlphaFold. Nature.

[80] Mirdita M., Schutze K., Moriwaki Y., Heo L., Ovchinnikov S., Steinegger M. (2022). ColabFold: making protein folding accessible to all. Nat. Methods.

[81] Hagan C.L., Kahne D. (2011). The reconstituted Escherichia coli Bam complex catalyzes multiple rounds of beta-barrel assembly. Biochemistry.

[82] Robert V., Volokhina E.B., Senf F., Bos M.P., Van Gelder P., Tommassen J. (2006). Assembly factor Omp85 recognizes its outer membrane protein substrates by a species-specific C-terminal motif. Plos Biol..

[83] Hagan C.L., Westwood D.B., Kahne D. (2013). Bam Lipoproteins Assemble BamA in vitro. Biochemistry.

[84] Webb C.T., Selkrig J., Perry A.J., Noinaj N., Buchanan S.K., Lithgow T. (2012). Dynamic association of BAM complex modules includes surface exposure of the lipoprotein BamC. J. Mol. Biol..

