# Supplementary material for: In vitro reconstitution implicates multiple OMP assembly factors in CdiAEC93 toxin delivery
Source: J Biol Chem. 2026 Sep 22;302(10):113512. doi: 10.1016/j.jbc.2026.113512 (PMC13625785; doi:10.1016/j.jbc.2026.113512)
Supplement: Supplementary file 1 [file mmc1.pdf]

In vitro reconstitution implicates multiple OMP assembly factors in CdiA^{EC93} toxin delivery

Ana Katrina Y. Tiu, Haley E. Murphy, Gabrielle M. Beal, Christine L. Hagan*

Supporting Information

Materials and Equipment

Cultures were grown in LB medium from BD Biosciences (cat. No. 214906) and Terrific Broth from Millipore Sigma (cat. No. 71754). Protein expression was induced with IPTG, L-arabinose, and L-rhamnose from Millipore Sigma (cat. No. I5502, A3256, and W373011, respectively). The BugBuster Protein Extraction Reagent, complete ULTRA EDTA-free protease inhibitor tablets, deoxyribonuclease I, lysozyme, PMSF, restriction grade thrombin, and HRV-3C protease were also purchased from Millipore Sigma (cat. No. 70584-3, 05892791001, DN25, L6876, P7626, 69671, and SAE0045, respectively). Trypsin was purchased from New England BioLabs (cat. No. P8101S). TCEP hydrochloride was purchased from Hampton Research (cat. No. HR2-801), and *p*-benzoylphenylalanine (*p*BPA) was purchased from Cayman Chemical (cat. No. 36168). The detergents, n-dodecyl-N,N-dimethylamine-N-oxide (DDAO) and n-dodecyl-β-D-maltoside (DDM) were purchased from Anatrace (cat. No. D360 and D310, respectively). The following phospholipids were purchased from Avanti Research as dried powders: *E. coli* polar lipid extract (cat. No. 100600), 1,2-didecanoyl-sn-glycero-3-phospho-(1'-rac-glycerol) (10:0 PG, cat. No. 840434), 1,2-didecanoyl-sn-glycero-3-phosphoethanolamine (10:0 PE, cat. No. 850700), 1,2-didecanoyl-sn-glycero-3-phosphocholine (10:0 PC, cat. No. 850325), 1,2-dilauroyl-sn-glycero-3-phosphocholine (12:0 PC, cat. No. 850335), 1,2-dimyristoyl-sn-glycero-3-phosphocholine (14:0 PC, cat. No. 850345), 1,2-dipalmitoyl-sn-glycero-3-phosphocholine (16:0 PC, cat. No. 850355), 1,2-distearoyl-sn-glycero-3-phosphocholine (18:0 PC, cat. No. 850365), 1,2-dioleoyl-sn-glycero-3-phosphocholine (DOPC, cat. No. 850375), and 1-palmitoyl-2-oleoyl-glycero-3-phosphocholine (POPC, cat. No. 850457).

The Ni-NTA Superflow resin was purchased from Qiagen (cat. No. 30430), and size-exclusion chromatography was performed on Superose 6 Increase 10/300 GL and Superdex 200 Increase 10/300 GL prepacked columns from GE Healthcare. Protein concentrations were determined using the Bio-Rad DC Protein Assay (cat. No. 500-0112).

SDS-PAGE was carried out on 4-20% TGX Stain-free gels from Bio-Rad (cat. No. 4568096 and 4568026). Immunoblotting was performed using low fluorescence PVDF from Bio-Rad (cat. No. 10026934) and a DyLight 680-labeled anti-Flag monoclonal antibody from Invitrogen (cat. No. MA1-91878-D680), a CF 488a-labeled anti-6X His monoclonal antibody from Millipore Sigma (cat. No. SAB4600048), and a Dylight 488-labeled goat anti-rabbit antibody from ThermoFisher (cat. No. 35553). The rabbit anti-BamA antibody, which recognizes the BamA POTRA domains, was a generous gift from Daniel Kahne. All gels and blots were imaged on a Bio-Rad ChemiDoc MP system.

Plasmid Construction

The plasmids used in this study are described in Table S1. The *E. coli* EC93 *cdiA* and *cdiBAI* sequences were amplified from pDAL661, which was a generous gift from David Low, and the *bamA*, *surA*, and *skp* sequences were amplified from *E. coli* MC4100 genomic DNA (11). The pET-21d, pET-22b, pET-26b, and pET-28b vectors were obtained from Novagen (cat. No. 69744, 69865, and 69743, respectively), and oligonucleotides were purchased from Eton BioScience and Integrated DNA Technologies. DNA sequences were amplified with KOD Hot

Start DNA polymerase (Millipore Sigma cat. No. 71086) and assembled with HiFi DNA Assembly Master Mix (New England BioLabs cat. No. E2621). The pCH103 plasmid, which encodes the mature sequence of BamA (without its signal sequence), and the pCH566 and pCH1145 plasmids, which carry the *E. coli* EC93 *cdiBAI* gene cassette, have been described previously (26, 31, 83). The plasmids encoding all five Bam proteins, pCH871 and pCH1275, were generated from pDT396, which was a gift from Daniel Kahne (51). The pEVOL-pBpF plasmid was a gift from Peter Schultz and was obtained from Addgene (plasmid #31190) (72, 73, 74). All DNA sequences were confirmed by Sanger sequencing or whole plasmid sequencing (Psomagen and Genewiz) and are available upon request.

Table S1. Plasmids used in this study

Name	Description
pCH1237	pET-28b-Flag-CdiA(R1358-K3132 ΔP1567-N1820)-His$_6$
pCH1240	pET-28b-Flag-CdiA(R1358-K3132 ΔP1567-N1820 W1519A)-His$_6$
pCH1241	pET-28b-Flag-CdiA(R1358-K3132 ΔP1567-N1820 N2908A)-His$_6$
pCH740	pET-22b-His$_6$-BamA(A21-W810)
pCH1230	pET-22b-His$_6$-BamA(A21-W810 Y550N/E554Q/H555Y)
pCH746	pET-22b-His$_6$-BamA(A21-W810 C690S/C700S)
pCH747	pET-22b-His$_6$-BamA(A21-W810 E435C/S665C/C690S/C700S)
pCH748	pET-22b-His$_6$-BamA(A21-W810 G393C/G584C/C690S/C700S)
pCH1189	pET-22b-His$_6$-BamA(A21-W810 D500C/S665C/C690S/C700S)
pCH1197	pET-22b-His$_6$-BamA(A21-W810 S559C/Q678C/C690S/C700S)
pCH1212	pET-22b-His$_6$-BamA(A21-W810 T659C/C690S/C700S/V713C)
pCH871	pTrc99a-BamA-BamB-BamC-BamD-BamE-His$_8$
pCH1275	pTrc99a-BamA(R76C)-BamB-BamC-BamD-BamE-His$_8$
pCH917	pET-28b-His$_6$-thrombin site-SurA(A21-N428)
pCH1272	pET-28b-His$_6$-thrombin site-SurA(A21-N428 K27C)
pCH1284	pET-28b-His$_6$-thrombin site-SurA(A21-N428 K27C/Q58*p*BPA)
pEVOL-pBpF	p15a-*M. jannaschii p*BPA tRNA synthetase and tRNA (Addgene plasmid #31190)
pCH01	pET-28b-His$_6$-thrombin site-Skp(A21-K161)
pCH566	pET-21dΔlac-CdiB-CdiA(Q643_G644ins3xFlag)-CdiI
pCH1145	pET-21dΔlac-CdiB-CdiA(Q643_G644ins3xFlag N2908A)-CdiI
pCH1295	pET-26b-SurA
pCH1309	pET-26b-Skp
pCH1313	pET-26b-MBP
pCH103	pET-22b-BamA(E22-W810)
pCH1183	pET-22b-BamA(E22-W810 Y550N/E554Q/H555Y))
pCH933	pET-28b-CdiA(R1358-G1573)-His$_6$
pCH1032	pET-28b-CdiA(R1358-G1573 W1519A)-His$_6$
pCH936	pET-28b-His$_6$-HRV-3C site-CdiA(R1358-G1573)

Supplemental Figures

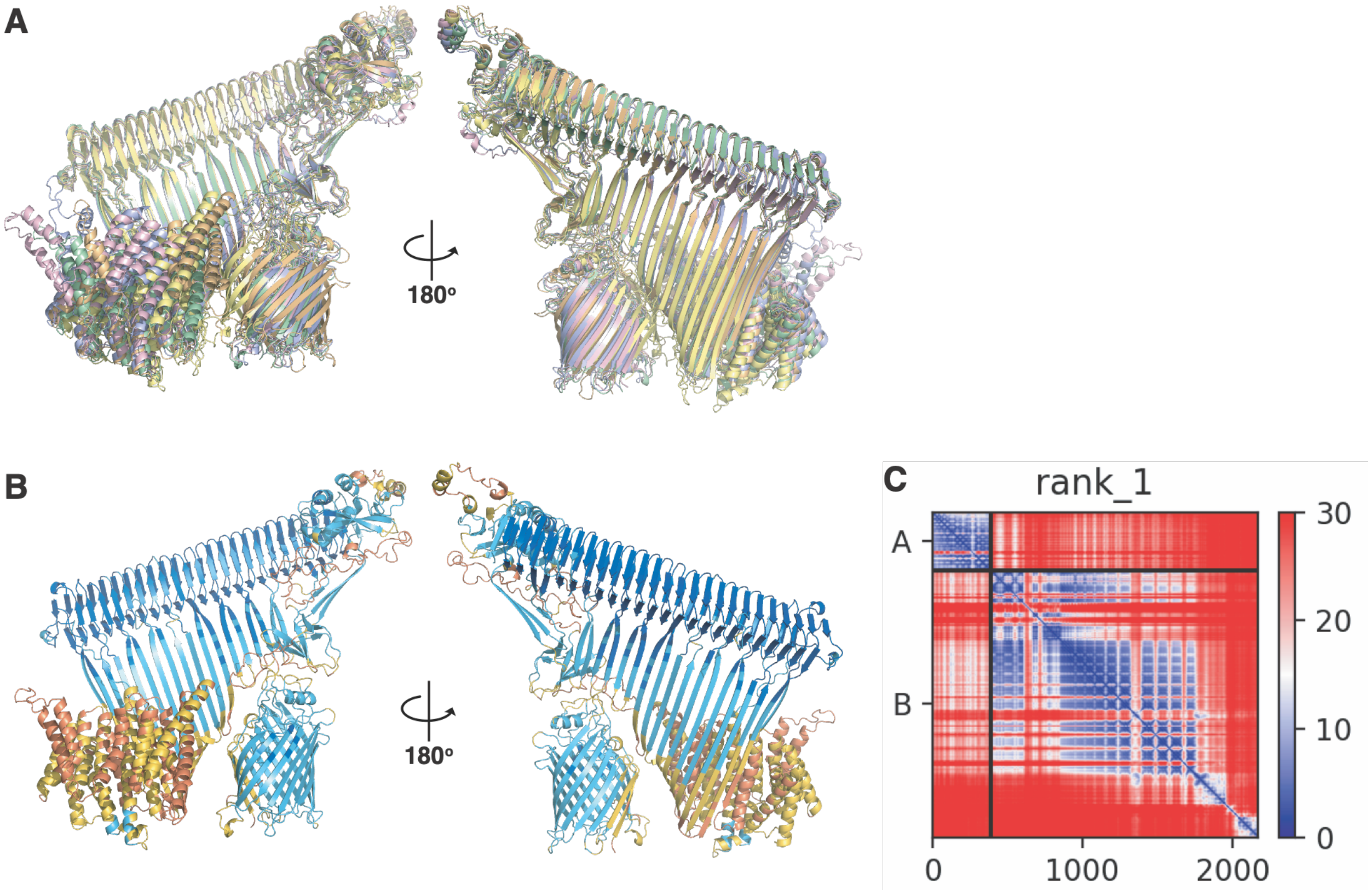

Figure S1. AlphaFold2 models of CdiA (amino acids R1358-K3132) with the BamA β-barrel (amino acids N422-W810) are largely consistent but display variability in the structure of the CdiA CT domain. **A.** Overlaid structures of five models of the CdiA-BamA complex generated by AlphaFold2 multimer using ColabFold. **B.** The top ranked model (as shown in Figure 1C) colored by pLDDT score (dark blue: pLDDT > 90, light blue: 90 > pLDDT > 70, yellow: 70 > pLDDT > 50, orange: pLDDT < 50). **C.** Predicted aligned error (PAE) of the top ranked model. The colored scale bar on the right depicts the PAE score in Angstroms. Protein A is the BamA β-barrel sequence (N422-W810) and protein B is the CdiA sequence (R1358-K3132).

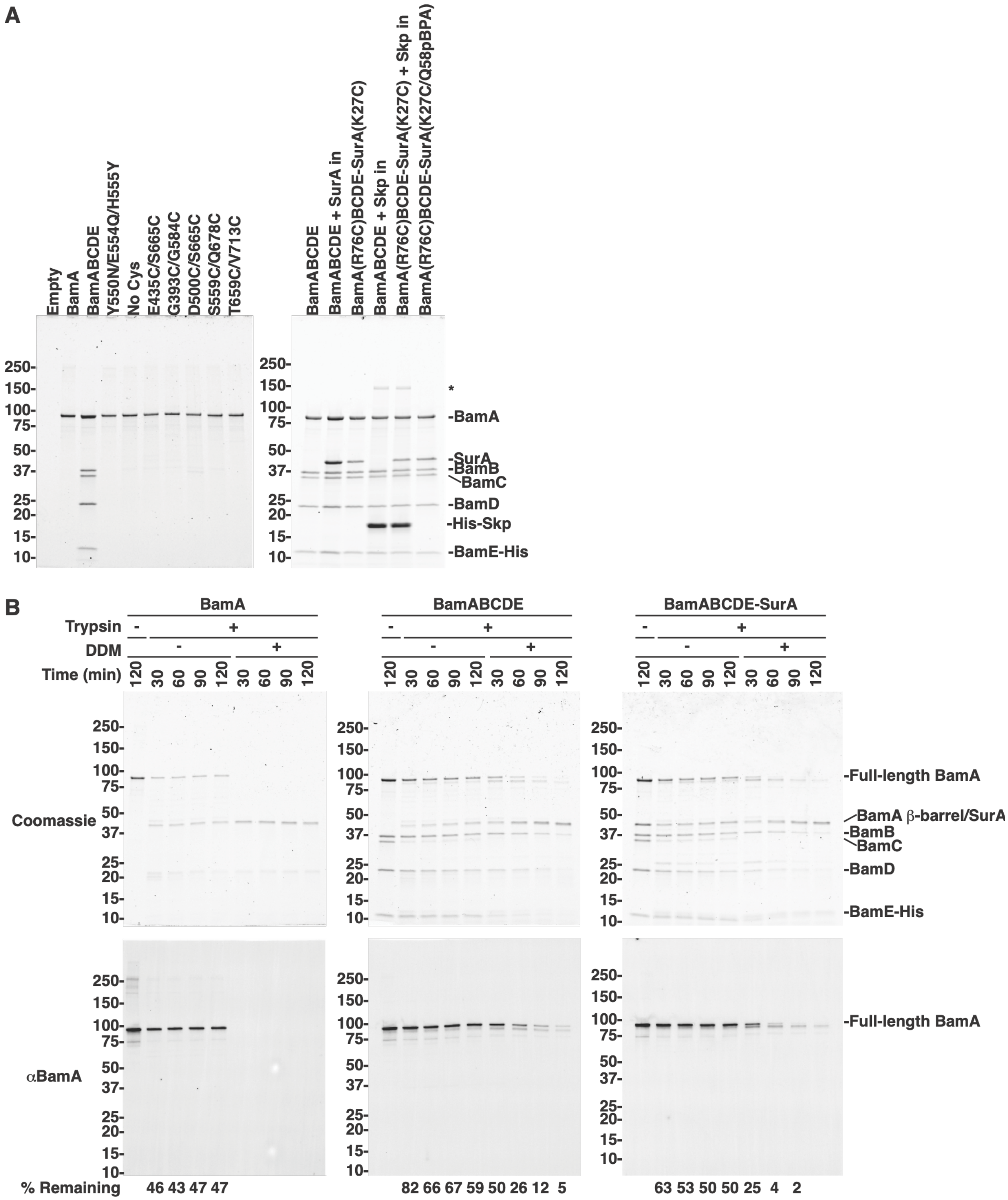

Figure S2. Analysis of the proteoliposomes used in this study. **A.** Reducing SDS-PAGE analysis of the proteoliposomes in this study indicates that they contain approximately equal amounts of the expected Bam proteins. The proteoliposomes were all prepared with 10 μM solutions of the BamA proteins or Bam complexes. His-Skp appears to be present at a higher concentration because it functions as a trimer and was therefore added at a concentration of 10 μM based on the

molecular weight of the trimeric form (*i.e.*, 30 μM of the monomer). A minor contaminant (indicated by *), which does not appear to interfere with the assays in this study, is present in the His-Skp preparation. **B.** A substantial percentage of the BamA proteins in the proteoliposome membranes are in the correct orientation to interact with CdiA. Proteoliposomes containing BamA, BamABCDE, and BamABCDE-SurA complexes were digested with trypsin in the presence or absence of 1% (w/v) DDM. When the proteoliposomes are intact, trypsin degrades the BamA molecules that have their N-terminal POTRA domains exposed, yielding a stable BamA fragment with a molecular weight of approximately 43 kDa (82). The remaining full-length BamA proteins are not degraded likely because they are oriented with their POTRA domains in the lumen of the proteoliposomes. When the proteoliposomes membranes are disrupted with DDM, all (or nearly all) of the BamA molecules and the BamB, D, and E lipoproteins become susceptible to digestion and are degraded over time. BamC is known to be susceptible to protease digestion even when it is internally localized (84). The percentage of full-length BamA remaining in each sample was calculated by comparing the intensity of the band in the digested (+ trypsin) sample to that in the undigested (- trypsin) sample in each anti-BamA immunoblot. The degraded BamA fragments do not appear in the immunoblots because the anti-BamA antibodies recognize the POTRA domains.

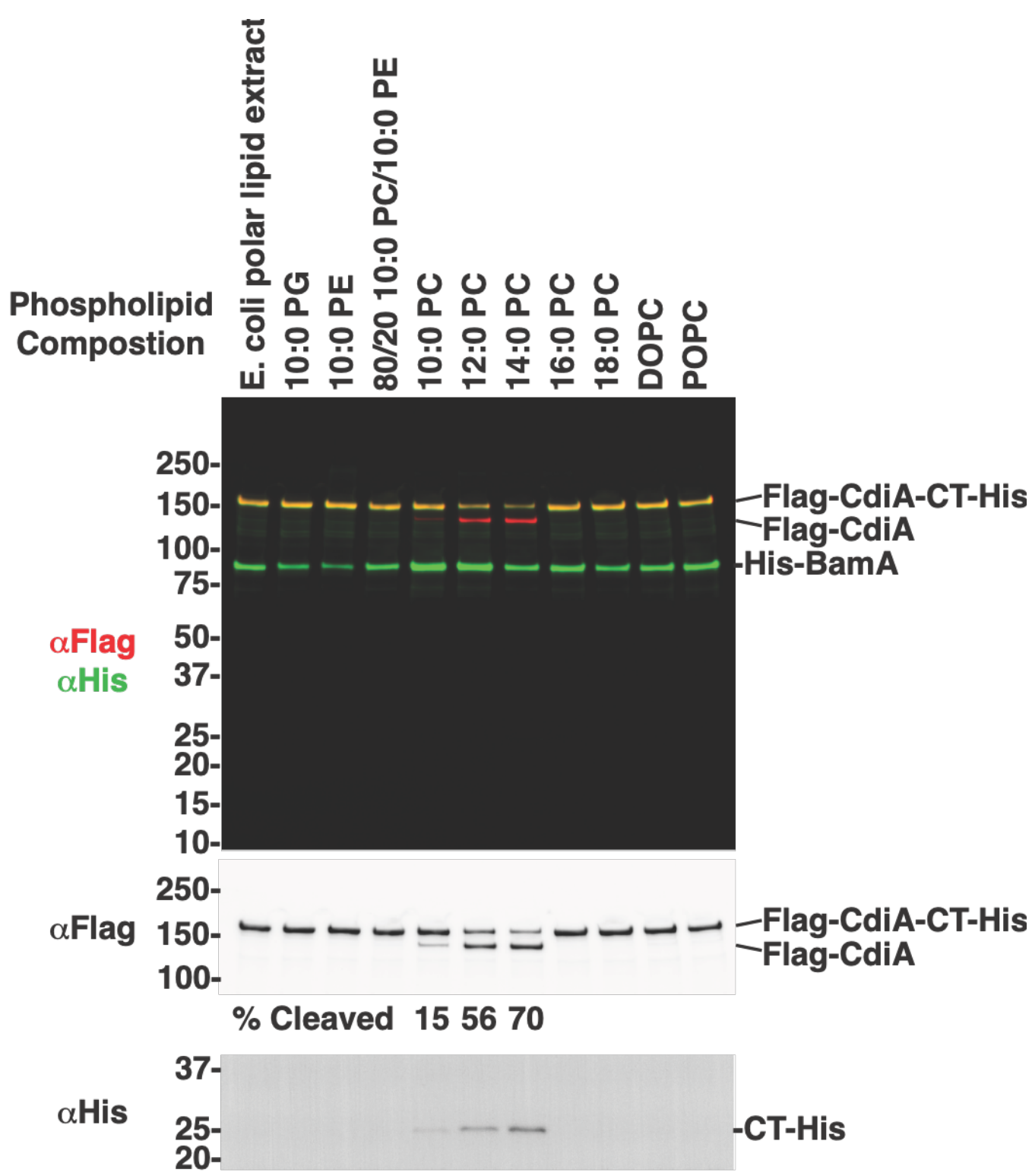

Figure S3. The Flag-CdiA-CT-His fragment cleaves most efficiently in BamA proteoliposomes composed of DMPC (14:0 PC) lipids. Proteoliposomes were prepared with the indicated phospholipids and purified His-BamA. Flag-CdiA-CT-His was added to each of these proteoliposomes and incubated at 25 ºC for four hours. The efficiency of Flag-CdiA-CT-His cleavage was determined by SDS-PAGE and immunoblotting with anti-Flag DyLight 680 (red in top panel and grayscale in middle panel) and anti-His CF 488 A (green in top panel and grayscale in bottom panel) antibodies.

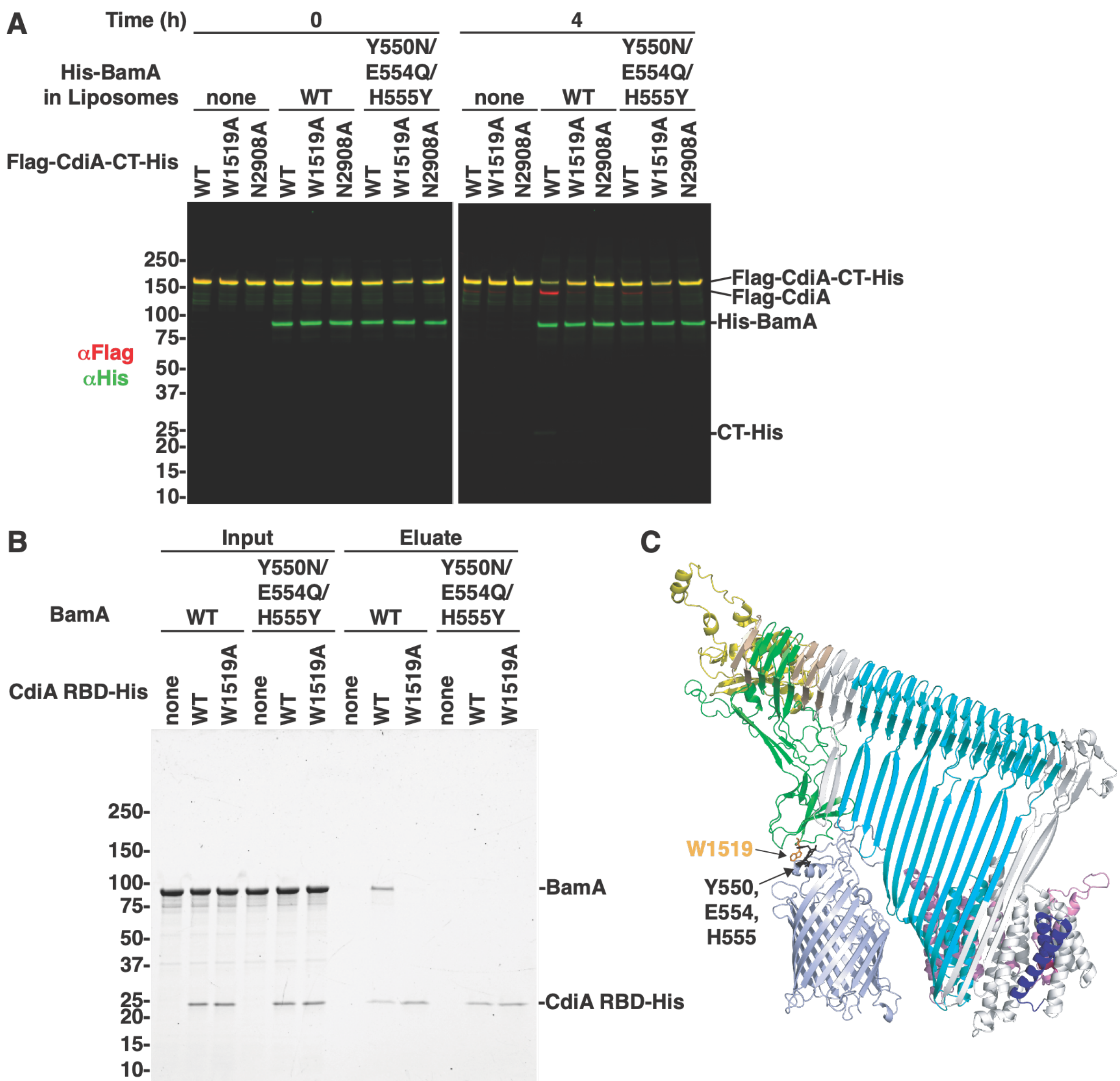

Figure S4. Amino acid substitutions that disrupt the interaction between BamA's extracellular loops and CdiA's RBD reduce the efficiency of CdiA toxin release. **A.** The full immunoblots of the experiment in Figure 3A are depicted with the bands detected by the anti-Flag DyLight 680 (red) and anti-His CF 488A (green) antibodies. **B.** In vitro co-purification of wild-type and altered forms of BamA with the CdiA RBD (amino acids R1358-G1573). Purified BamA and CdiA RBD carrying a C-terminal His_6-tag were mixed in a 3:1 molar ratio and then subjected to Ni-affinity purification. The input and eluate samples were analyzed by SDS-PAGE and Coomassie staining. The wild-type CdiA RBD pulls down wild-type BamA but not the Y550N/E554Q/H555Y BamA, and the W1519A CdiA RBD does not pull down either form of BamA. **C.** AlphaFold2 structure of the CdiA-BamA complex depicting the positions of the altered amino acids in the experiments in **A** and **B**. W1519 in the CdiA RBD is shown in orange, and Y550, E554, and H555 in BamA's extracellular loop 4 are shown in black.

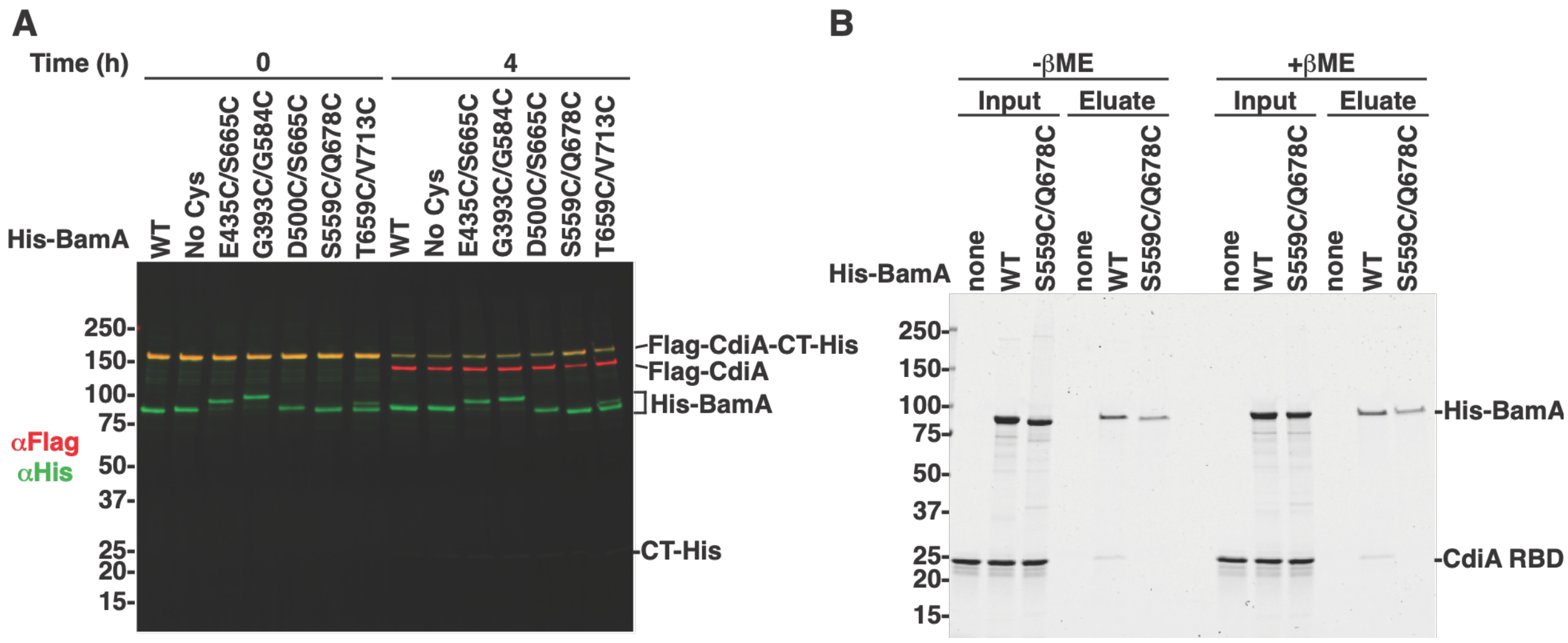

Figure S5. A disulfide crosslink between L4 and L6 in BamA reduces the efficiency of CdiA toxin release in vitro by reducing the binding affinity for the CdiA RBD. **A.** The full immunoblot of the experiment in Figure 3D is shown with the bands detected by the anti-Flag DyLight 680 and anti-His CF 488A antibodies shown in red and green, respectively. The His-BamA proteins have different apparent molecular weights because the samples were analyzed under non-reducing conditions such that the internal disulfide bonds were maintained during SDS-PAGE. **B.** The CdiA RBD does not co-purify with S559C/Q678C His-BamA in vitro. Purified CdiA RBD was mixed with His-tagged BamA (wild-type or S559C/Q678C) in a 3:1 ratio and then subjected to Ni-affinity purification. The input and eluate samples were analyzed by SDS-PAGE under non-reducing [*i.e.*, without β-mercaptoethanol (-βME)] and reducing (+βME) conditions and Coomassie stained.

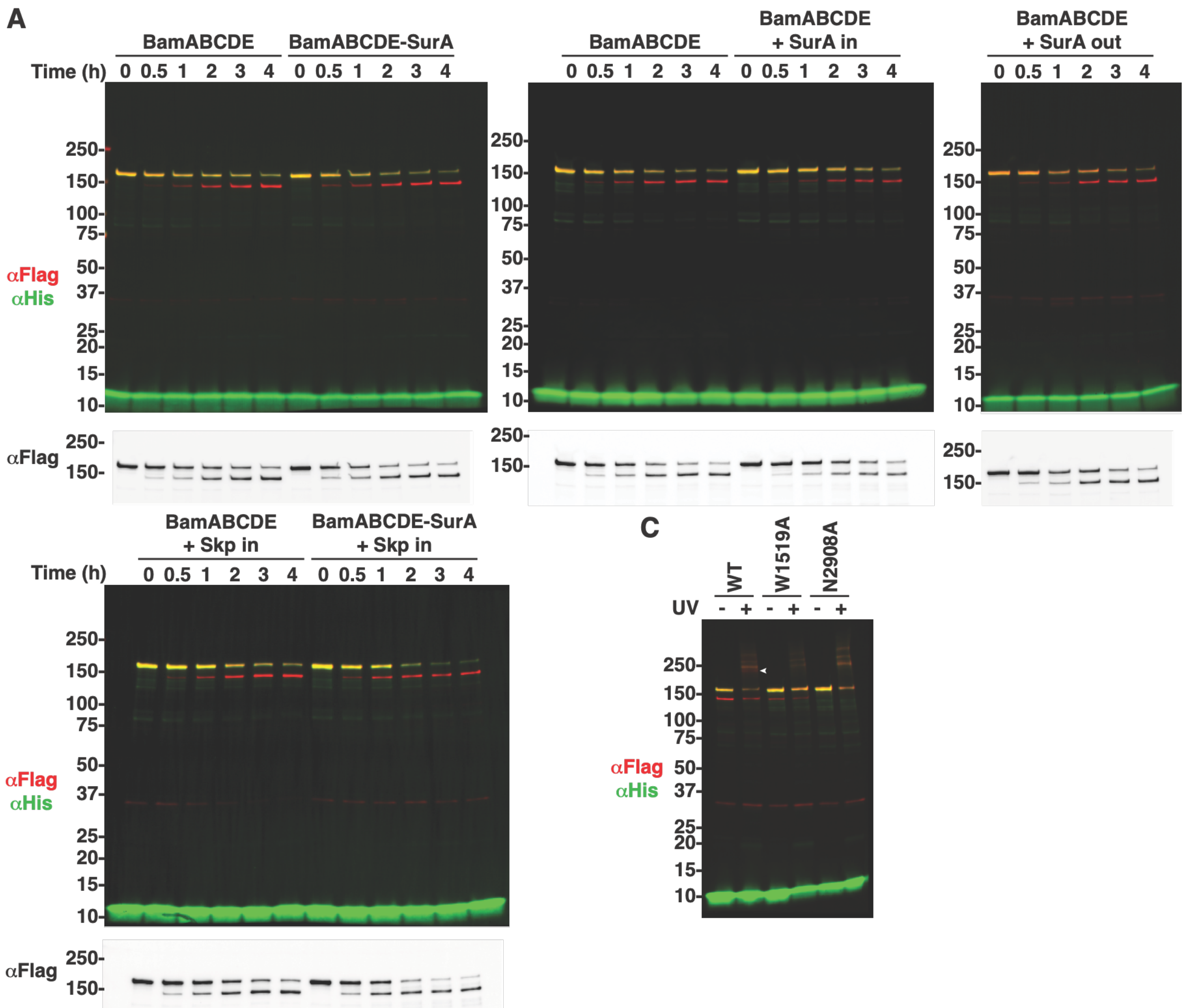

B

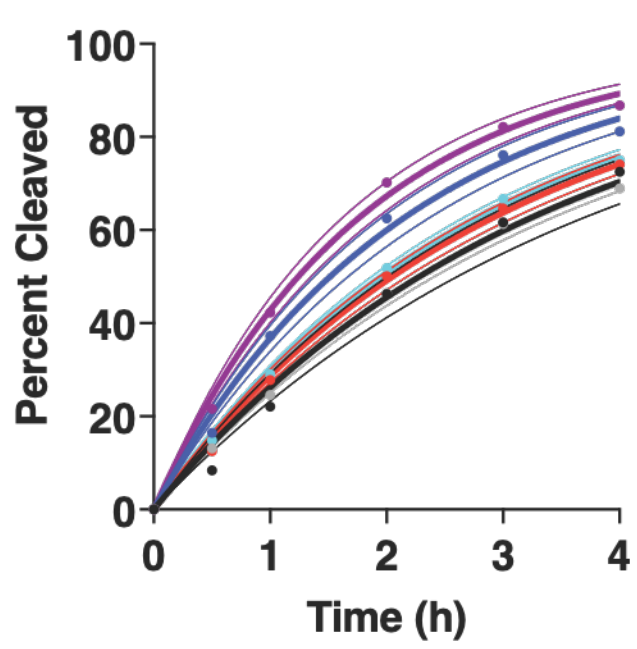

Proteoliposome Contents	CdiA Cleavage $t_{1/2}$ (h)	95% Confidence Interval (h)	Fold Change in $t_{1/2}$ Relative to BamABCDE
BamABCDE	2.280	2.005 – 2.601	-
BamABCDE + SurA out	2.051	1.934 – 2.175	1.112
BamABCDE + SurA in	2.289	2.176 – 2.409	0.996
BamABCDE-SurA	1.514	1.374 – 1.667	1.506
BamABCDE + Skp in	1.950	1.873 – 2.031	1.169
BamABCDE-SurA + Skp in	1.241	1.140 – 1.350	1.837

Figure S6. SurA and Skp increase the rate of CdiA toxin release in vitro. **A.** Representative immunoblots of CdiA cleavage over time in proteoliposomes containing the BamABCDE complex and SurA and Skp. The bands detected by anti-Flag DyLight 680 and anti-His CF 488A antibodies shown in red and green, respectively, in the top panels. The His-tagged BamE is detected at the expected molecular weight of ~11 kDa. The cleaved (Flag-CdiA) and uncleaved (Flag-CdiA-CT-His) bands are shown in grayscale in the lower panels; the percentage of cleaved CdiA at each time point was determined by comparing the intensities of these bands. The data from these

experiments and two additional replicates are plotted in Figure 5A. **B.** SurA and Skp increase the rate of CdiA cleavage by a small but significant amount. The data in Figure 5A were fit with one phase association curves (thick lines), and the 95% confidence interval for each curve is displayed (thin lines). The curves are colored as in Figure 5A: BamABCDE in black, BamABCDE + SurA out in red, BamABCDE + SurA in in gray, BamA(R76C)BCDE-SurA(K27C) in dark blue BamABCDE + Skp in in cyan, and BamA(R76C)BCDE-SurA(K27C) + Skp in in purple. The calculated half-lives of Flag-CdiA-CT-His cleavage in each of the proteoliposomes are listed in the table with their 95% confidence intervals. **C.** The full immunoblot of the experiment in Figure 5B is shown with the bands detected by the anti-Flag DyLight 680 and anti-His CF 488A antibodies shown in red and green, respectively. Most of the CdiA-SurA adducts appear yellow because they are recognized by both antibodies, indicating that the Flag-CdiA-CT-His crosslinked to SurA before it was cleaved, but the white arrow indicates one adduct which appears red because it lacks its C-terminal His-tag.

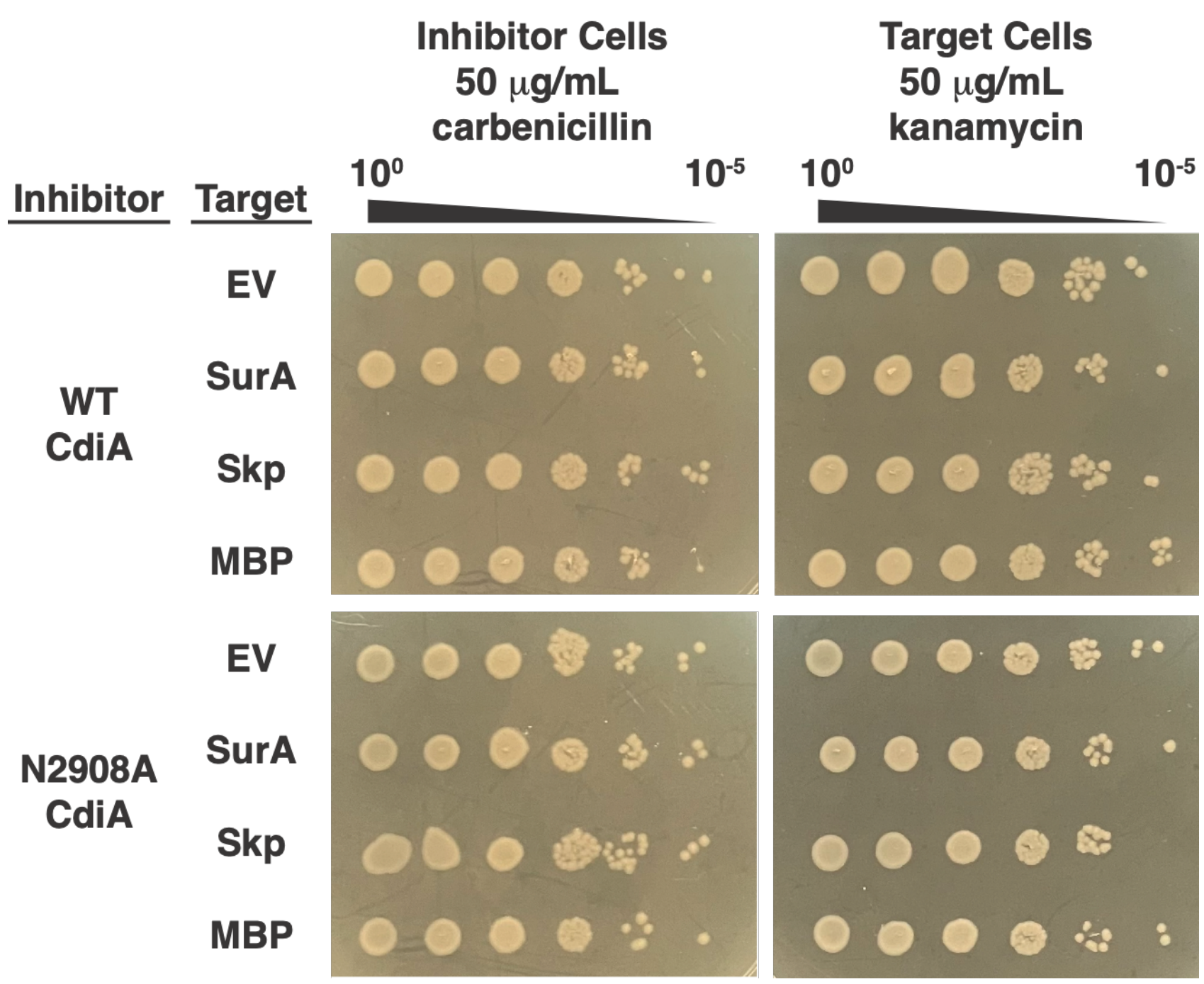

Figure S7. Inhibitor and target cells were present in equal numbers at the beginning of the co-culture experiment. Samples of the co-cultures from the experiment in Figure 6 were removed and serially diluted immediately after the inhibitor and target cells were mixed and protein expression was induced. The dilutions were plated on selective media to assess the number of colony-forming units of the inhibitor (carbenicillin-resistant) and target (kanamycin-resistant) cells present in the mixtures.
